# Impact of airway closure and lung collapse on inhaled nitric oxide effect in acute lung injury: an experimental study

**DOI:** 10.1186/s13613-024-01378-z

**Published:** 2024-09-23

**Authors:** Mariangela Pellegrini, Mayson L. A. Sousa, Sebastian Dubo, Luca S. Menga, Vanessa Hsing, Martin Post, Laurent J. Brochard

**Affiliations:** 1 Anesthesiology and Intensive Care Medicine, Uppsala University Hospital, Uppsala, Sweden; 2 Hedenstierna Laboratory, Department of Surgical Sciences, Uppsala University Hospital, Akademiska sjukhuset, ing 40 2 tr. 751 85, Uppsala, Sweden; 3 Keenan Centre for Biomedical Research, Critical Care Department, St. Michael’s Hospital, Unity Health Toronto, Toronto, Canada; 4 Interdepartmental Division of Critical Care Medicine, University of Toronto, Toronto, Canada; 5 Translational Medicine Program, Peter Gilgan Centre for Research and Learning, The Hospital for Sick Children, Toronto, Canada; 6 Department of Physiotherapy, Universidad de Concepción, Concepción, Chile; 7 Department of Physiology, University of Toronto, Toronto, Canada

**Keywords:** (3 to 10) nitric oxide, Acute respiratory distress syndrome, Mechanical ventilation, Airway closure

## Abstract

**Background:**

Efficacy of inhaled therapy such as Nitric Oxide (iNO) during mechanical ventilation may depend on airway patency. We hypothesized that airway closure and lung collapse, countered by positive end-expiratory pressure (PEEP), influence iNO efficacy. This could support the role of an adequate PEEP titration for inhalation therapy. The main aim of this study was to assess the effect of iNO with PEEP set above or below the airway opening pressure (AOP) generated by airway closure, on hemodynamics and gas exchange in swine models of acute respiratory distress syndrome. Fourteen pigs randomly underwent either bilateral or asymmetrical two-hit model of lung injury. Airway closure and lung collapse were measured with electrical impedance tomography as well as ventilation/perfusion ratio (V/Q). After AOP detection, the effect of iNO (10ppm) was studied with PEEP set randomly above or below regional AOP. Respiratory mechanics, hemodynamics, and gas-exchange were recorded.

**Results:**

All pigs presented airway closure (AOP > 0.5cmH_2_O) after injury. In bilateral injury, iNO was associated with an improved mean pulmonary pressure from 49 ± 8 to 42 ± 7mmHg; (*p* = 0.003), and ventilation/perfusion matching, caused by a reduction in pixels with low V/Q and shunt from 16%[IQR:13–19] to 9%[IQR:4–12] (*p* = 0.03) only at PEEP set above AOP. iNO had no effect on hemodynamics or gas exchange for PEEP below AOP (low V/Q 25%[IQR:16–30] to 23%[IQR:14–27]; *p* = 0.68). In asymmetrical injury, iNO improved pulmonary hemodynamics and ventilation/perfusion matching independently from the PEEP set. iNO was associated with improved oxygenation in all cases.

**Conclusions:**

In an animal model of bilateral lung injury, PEEP level relative to AOP markedly influences iNO efficacy on pulmonary hemodynamics and ventilation/perfusion match, independently of oxygenation.

**Supplementary Information:**

The online version contains supplementary material available at 10.1186/s13613-024-01378-z.

## Background

Acute respiratory distress syndrome (ARDS) is a life-threatening lung condition associated with high morbidity and mortality [1, 2]. ARDS is characterized by an inflammatory process of the alveolar-capillary membrane, diffuse micro-thrombosis and late fibrotic evolution [3, 4], all leading to a ventilation-perfusion (V/Q) mismatch, increased right heart afterload, and subsequent deranged gas exchange [5, 6]. Complete airway closure - the premature closing of the airways during expiration with distal lung parenchyma still aerated - is prevalent in 30 to 60% of mechanically ventilated ARDS patients. Airway closure can happen both peripherally and centrally in the lung parenchyma. When happening centrally, it is possible to spirometrically detect an airway opening pressure (AOP) by performing a low-flow inflation maneuver and, along with lung collapse – the deflation and closure of distal lung parenchyma - may be an important contributor to V/Q mismatch in this population [7, 8]. It has also been described in cardiogenic pulmonary edema or in obese patients during surgery [9, 10]. Airway closure can be detected by performing a low-flow insufflation maneuver, and identifying the airway opening pressure (AOP) during quasi-static conditions. It can also be suspected from the airway pressure profile during volume control ventilation [11]. In addition, AOP can vary across different lung regions [12]. Setting positive end-expiratory pressure (PEEP) above the AOP can prevent repeated airway closure and reopening, maintain airway patency and limit reabsorption atelectasis [13]. We reasoned that airway closure can also affect the delivery of inhaled therapies [14] and PEEP could promote the efficacy of any inhaled therapy.

To date, protective ventilation (i.e., low driving pressure and limited tidal volume [15, 16]) is the main approach proven to improve ARDS mortality, while pharmacological therapies lack well-established benefits. Considering its selective vasodilatory effect in ventilated lung units and the consequent reduction in alveolar dead space and right heart afterload [17], two determinants of mortality in ARDS patients [18, 19], inhaled nitric oxide (iNO) offers interesting physiological effects in ARDS. Despite physiological evidence of iNO in reducing pulmonary pressure and improving oxygenation [20], its role in ARDS is still controversial, as no reported clinical trials and metanalyses have demonstrated clinical efficacy and benefits in terms of survival [21, 22]; nevertheless, iNO is still used as a rescue therapy by clinicians, as observed during the COVID-19 pandemic [23, 24]. Referring to the pharmacokinetics and the rheological characteristics of any inhaled gas therapy, a proper delivery of gas at the alveolar level requires patency of the airways [25]. Therefore, airway closure as well as lung collapse may negatively affect iNO efficacy in ARDS although this has not been studied. Airway closure may hinder the inhaled gases reaching the more distal airway, thereby limiting their therapeutical effect, and this hindrance may vary according to the distribution of lung injury. We hypothesize here that PEEP needs to be set above AOP to ensure the full effect of iNO therapy and thought this could give useful information for the use of any inhaled therapy. In this randomized experimental study, we assessed the influence of the distribution of lung injury (i.e., bilateral vs. asymmetrical injury) and PEEP level set below and above AOP on the efficacy of the iNO therapy in two swine models of ARDS. Efficacy was investigated regarding pulmonary hemodynamics, gas exchange and regional V/Q matching.

## Methods

This study followed the Canadian Animal Care guidelines and was approved by the local Animal Care Committee (AUP58058) of The Hospital for Sick Children, Toronto, Canada. For more detailed methods, see online supplemental material.

### Animal preparation and lung injury

Supine anesthetized and mechanically ventilated pigs underwent animal preparation. For monitoring of hemodynamics and blood sampling, a femoral PiCCO arterial line (Getinge, Sweden) and a pulmonary artery catheter (Edwards Lifesciences, USA) were used. For monitoring of respiratory mechanics, an esophageal balloon catheter was placed [26]. Airway flow and pressure were recorded at the airway opening, and tidal volume was calculated as integral of the flow. An Electrical Impedance Tomography (EIT) belt was placed to collect impedance changes related to ventilation and lung perfusion (Pulmovista 500, Dräger, Germany). Peripheral oxygen saturation was continuously monitored (Siemens Healthineers, Germany). Animals were randomized between two models of ARDS (i.e., asymmetrical or bilateral lung injury), induced by a two-hit injury (surfactant lavage followed by high-stretch ventilation) targeted to one or two lungs respectively, as previously described [27, 28], (Figure E1). A low-flow inflation maneuver (5 L/min) to detect the presence of AOP was performed after lung injury, and after a prolonged expiration phase, and EIT-based AOP values [12] (Figure E2) were used to guide the interventions described below. Recruitability was assessed by the recruitment-to-inflation ratio [29].

### Intervention protocol and data collection

**Fig. 1:**
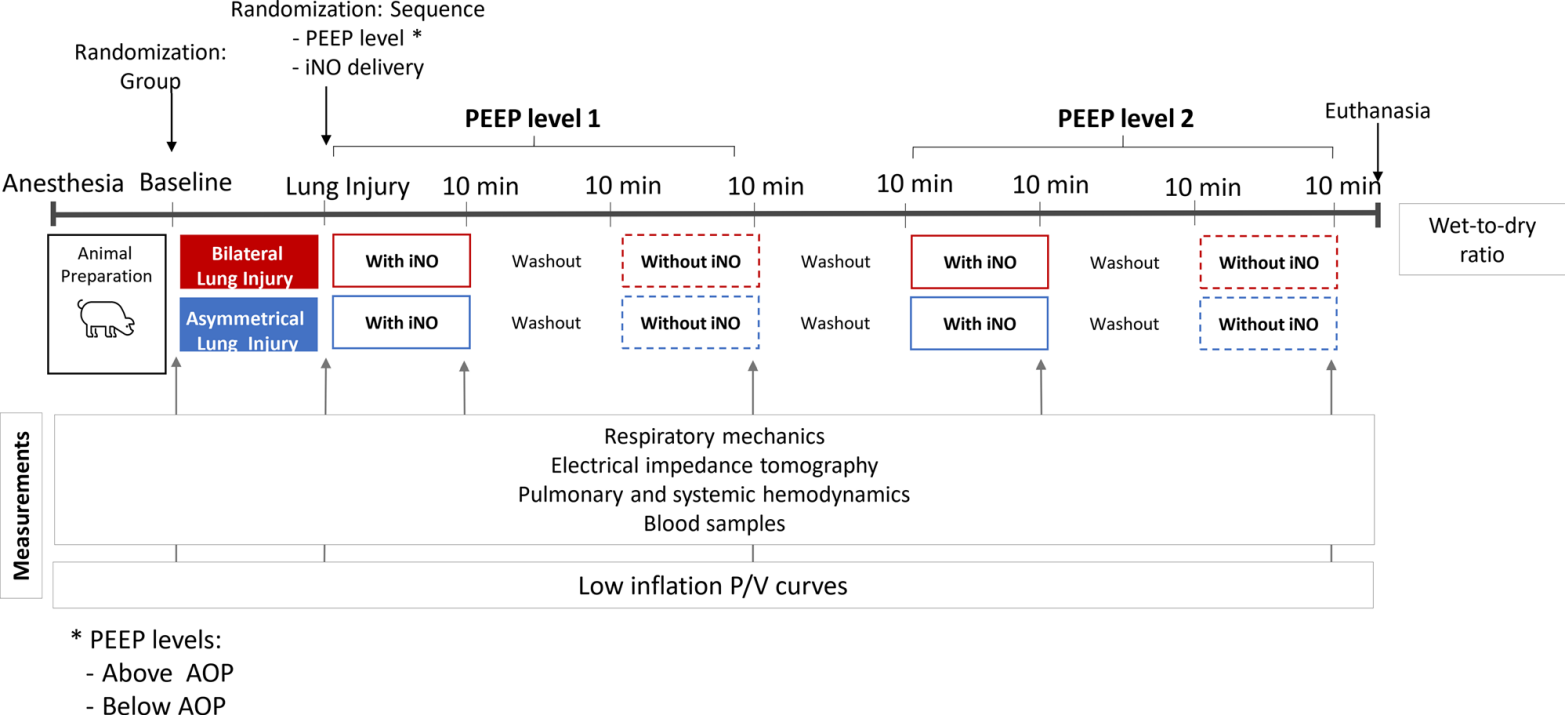
Study protocol overview. Abbreviations: PEEP, Positive End-Expiratory Pressure; iNO, inhaled nitric oxide; aop, airway opening pressure. *: PEEP levels above/below AOP; **: this flowchart represents a sequence of 10-min with iNO at 10 ppm followed by 10-min without iNO

**Fig. 2:**
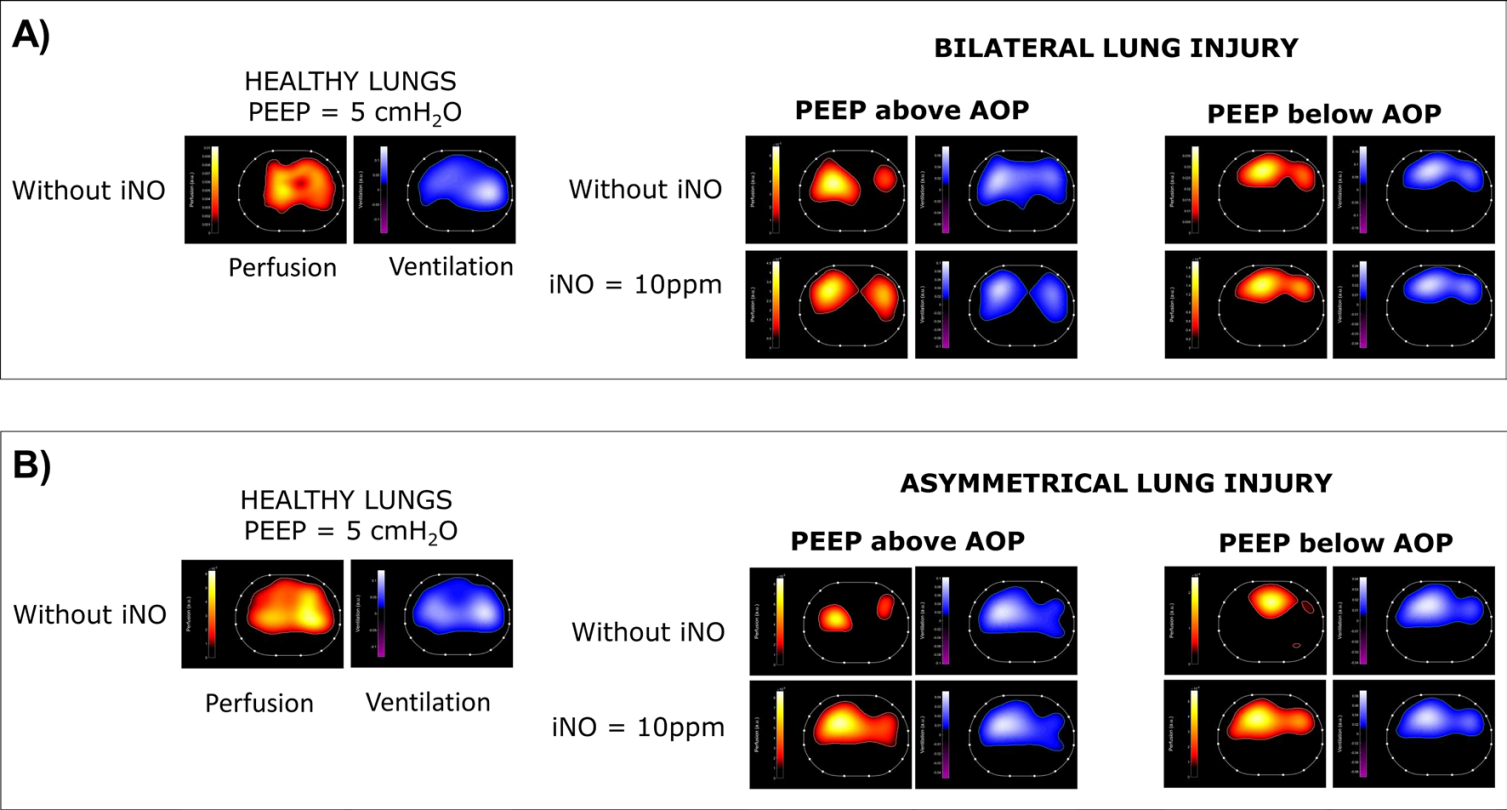
Electrical impedance tomography in a representative example of bilateral **(A)** and asymmetrical **(B)** lung injury. For each studied condition (i.e., healthy lung - HL, injured lung at PEEP above/below AOP, with/without iNO two derived maps are reported: *1) the perfusion map* reported in red-orange-yellow scale (where yellow represents high perfusion and red low perfusion); *2) the ventilation map* reported in blue-sky blue-white (where white corresponds to highly ventilated areas). Abbreviations: PEEP: positive end expiratory pressure; iNO: inhaled nitric oxide; AOP: airway opening pressure

All animals were ventilated with low-tidal volume (6–8 mL/kg) and two levels of PEEP: (1) PEEP above AOP, set 2 cmH_2_O above the highest regional AOP; (2) PEEP below AOP, set below 2 cmH_2_O (or less if AOP was lower than 2 cmH_2_O) below the lowest regional AOP. At each level of PEEP, the pigs were ventilated for 10 min without iNO and for 10 min with iNO at 10 ppm (Air Liquide Healthcare, Paris, France) administered by SoKINOX (INOsystems, Air Liquide Healthcare, Paris, France) before data acquisition. There was a “washout period” of 10 additional minutes between each step. This was to ensure enough equilibration of lung mechanics [30]. To minimize the influence of residual iNO during the zero iNO, in each experiment, we randomized the order of PEEP (i.e., below AOP vs. above AOP) and the order of iNO administration (i.e., with vs. without iNO). Pulmonary and systemic hemodynamics, gas exchange and respiratory mechanics were measured at baseline and, after lung injury, at the end of each studied condition (Fig. 1). Pulmonary artery pressure (PAP), central venous pressure, systemic arterial pressure, heart rate, airway pressure, airway flow, and esophageal pressure were acquired at 1 kHz in LabChart (ADInstruments, Australia). Cardiac output, extra-vascular lung water, and global end-diastolic volume were estimated by PiCCO, as an average of three measurements for each time point. Mixed venous and arterial blood samples were analyzed (Nova Biomedical, USA). Synchronized mechanical ventilation (Evita Infinity V500, Dräger, Germany) and EIT tracings were continuously recorded with a sample rate of 50 Hz. For the acquisition of ventilation and perfusion EIT maps, a 30-seconds respiratory-hold at mean airway pressure was performed and a 10 ml bolus of a hypertonic sodium chloride solution (5% NaCl) was centrally infused, as recommended by Dräger for Pulmovista 500 [31]. EIT data were subsequently processed offline by commercial software (EIT Perfusion Analysis SW v1.2.0, Dräger) to obtain 32 × 32 ventilation and perfusion EIT maps (Fig. 2). At the end of the experiment, the animals were euthanized with an overdose of pentobarbital. Samples of the dependent and non-dependent regions of each lung were collected to assess lung water accumulation by wet-to-dry ratio.

### Data analysis

The PAP was monitored and the transpulmonary vascular pressure gradient was calculated as mean pulmonary artery pressure (mPAP) minus wedge pressure. Pulmonary vascular resistance (PVR) was calculated as transpulmonary vascular pressure gradient divided by CO. EIT-derived ventilation and perfusion maps (Fig. 2) were analyzed at a pixel level using custom-made MatLab (vR2023b, MathWorks, USA) scripts. For both the ventilation and the perfusion maps, the impedance of each pixel was expressed as percentage of the global impedance, and the log(V/Q) was calculated pixelwise. Each log(V/Q) could vary between − 1 and 1 as a continuous variable. To plot the gaussian distribution as histograms, log(V/Q) data were rounded to their closest first decimal (i.e., -1, -0.9, -0.8, -0.7…). To plot the log(V/Q) distribution, log(V/Q) values lower than or equal to -1 (V/Q < 0.1) were classified as estimate of shunt and rounded to -1, whereas log(V/Q) values equal to or higher than 1 (V/Q > 10) were classified as estimate of dead space and rounded to + 1. Twenty-one log(V/Q) compartments were so defined and plotted (x-axis) against the mean pixel-based percentage of ventilation and perfusion (y-axis) [32]. The distribution curves were then fitted with a Gaussian curve, of which peak, kurtosis (as a measure of tailedness) and area under the curve were used to quantify changes in V/Q. Based on classical physiology [33, 34], a three-compartment model was applied to the log(V/Q) pixels values to define: (1) low V/Q and shunt with log(V/Q)<-0.3 (corresponding to V/Q < 0.5); (2) normal V/Q with log(V/Q) between − 0.3 and 0.3; (3) high V/Q and dead space with log(V/Q) equal or above 0.3 (corresponding to V/Q > 2).

### Statistical analysis

Categorical variables were presented as counts and proportions, and continuous variables were reported as mean and standard deviation or as median and interquartile range. Multivariate Imputation by Chained Equations was applied to handle missing values. The presence of airway closure and the respective AOP were measured for each lung. The proportion of airway closure was reported overall and stratified by lung (right, left) and by injury (bilateral, asymmetrical). The proportions were compared between groups using Chi-square test or Fisher’s exact test for small samples. Univariate analyses, to assess the impact of iNO (“with iNO” vs. “without iNO”), were performed using paired Student’s t-test or Wilcoxon signed-rank test, for continuous variables, and Chi-square test or Fisher’s exact test, for categorical variables. The Shapiro-Wilk test was used to assess normality. Multivariate analyses were performed using Mixed Analysis of Variance (Mixed ANOVA) for variables with statistical significance in the univariate analysis. In the Mixed ANOVA models, each dependent variable (e.g., mean PAP) was analyzed with subject as the random effect, lung injury group as the fixed (between-subjects) effect, and PEEP and iNO as within-subjects factors. All tests were two-tailed with type-I error (alpha) set at 0.05. The statistical analyses were performed using MatLab and R software (https://www.R-project.org/).

## Results

We included a total of 14 female Yorkshire pigs in this study. Animal characteristics and lung injury variables are presented in Table 1. The respiratory system static compliance (C_RS_) and the pressure of oxygen in arterial blood (PaO_2_) to the inspired oxygen fraction (F_I_O_2_) ratio (PaO_2_/FIO_2_) were lower and recruitment-to-inflation (R/I) ratio was higher in pigs with bilateral injury than in pigs with asymmetrical injury. Wet-to-dry ratio differed between the right (6.35 ± 1.05) and left (7.91 ± 1.23; *p* = 0.04) dependent regions in the asymmetrical group, while there was no difference in the bilateral group (right: 7.38 ± 2.60; left: 8.34 ± 0.66; *p* = 0.620). There were no differences between right and left wet-to-dry ratios in the non-dependent regions in both groups (Figure E3).

**Table 1:** Pig characteristics and induced lung injury

Variables	Total Sample (*n* = 14)	Asymmetrical Lung Injury (*n* = 7)	Bilateral Lung Injury (*n* = 7)	*p*
Weight (kg)	39 ± 4	41 ± 3	38 ± 4	0.227
Before Lung Injury				
C_RS_ (mL/cmH_2_O)	33 ± 8	32 ± 4	34 ± 10	0.701
PaO_2_/FiO_2_ (mmHg)	479 ± 55	456 ± 65	500 ± 40	0.192
R/I ratio	0.18 ± 0.07	0.17 ± 0.08	0.18 ± 0.05	0.969
Number of Lavages	5 ± 2	4 ± 1	6 ± 2	0.011
PaO_2_/FiO_2_ after lavages (mmHg)	78 ± 13	75 ± 12	81 ± 14	0.438
Final Lung Injury				
C_RS_ (mL/cmH_2_O)	18 ± 5	21 ± 3	15 ± 4	0.010
PaO_2_/FiO_2_ (mmHg)	89 ± 38	116 ± 35	61 ± 13	0.006
R/I ratio	0.76 ± 0.50	0.40 ± 0.30	1.12 ± 0.40	0.003

*Abbreviations* C_RS_, respiratory system compliance; PaO_2_/FiO_2_, ratio of arterial oxygen partial pressure (in mmHg) to fractional inspired oxygen; R/I ratio, recruitment-to-inflation ratio. *Notes*: Data are presented as mean and standard deviation. T-test or Mann-Whitney test for comparisons between two groups

### Airway closure

**Fig. 3:**
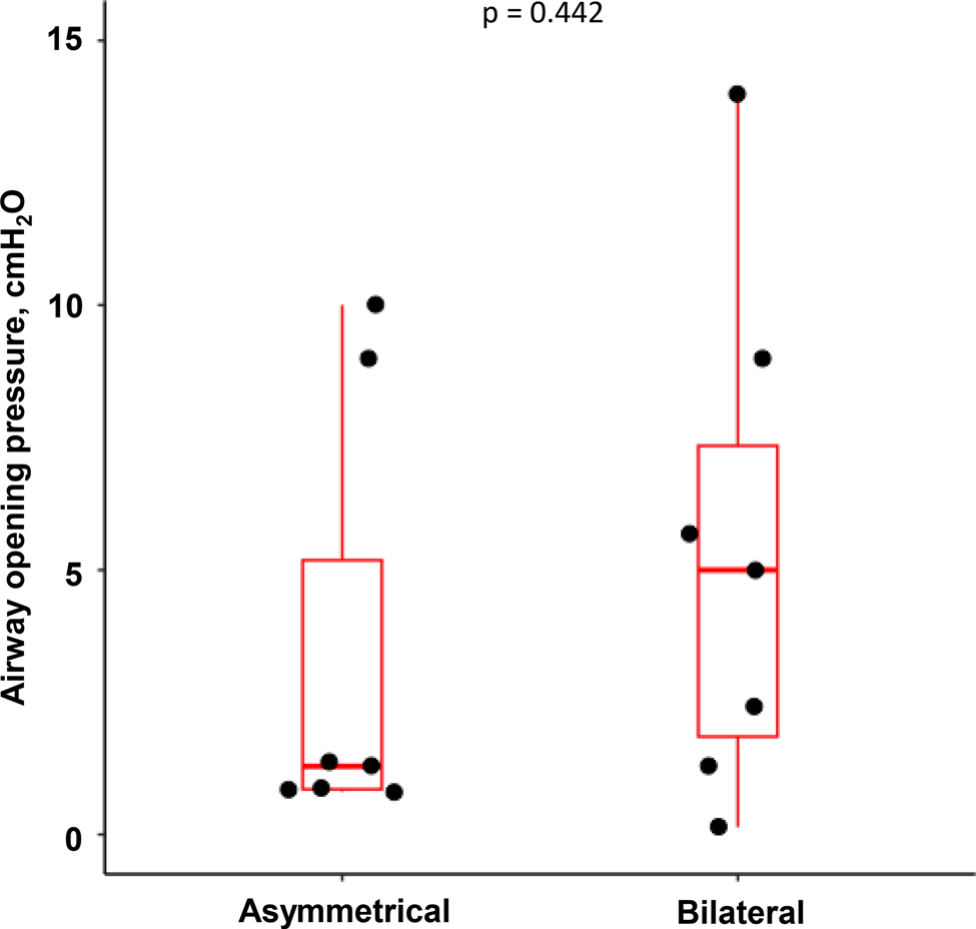
Highest global airway opening pressure (AOP) by group

All pigs exhibited some airway closure (global AOP > 0.5cmH_2_O) (Fig. 3) after lung injury though at relatively low values in several animals. Median and interquartile range (IQR) right and left AOP were, respectively, 5cmH_2_O (IQR:2-7cmH_2_O; Min:0.5cmH_2_O-Max:10cmH_2_O) and 2cmH_2_O (IQR:2-7cmH_2_O; Min:0.5cmH_2_O-Max:14cmH_2_O) in the bilateral group (*p* = 0.937), and 1cmH_2_O (IQR:1-2cmH_2_O; Min:0.6cmH_2_O-Max:3cmH_2_O) and 1cmH_2_O (IQR:1-5cmH_2_O; Min:0.8cmH_2_O-Max:10cmH_2_O) in the asymmetrical group (*p* = 0.653). The median and IQR of global AOP was 2cmH_2_O (IQR:1-7cmH_2_O; Min:0.5cmH_2_O-Max:10cmH_2_O) in the bilateral group and 1 cmH_2_O (IQR:1-2cmH_2_O) in the asymmetrical group, *p* = 0.609.

### Respiratory mechanics, lung volume and regional distribution of ventilation

The median values of selected PEEP above AOP were 8cmH_2_O (Min: 3cmH_2_O- Max: 15cmH_2_O), and 0cmH_2_O (Min: 0cmH_2_O - Max:10cmH_2_O) below AOP in the bilateral injury group. In bilateral injury, respiratory mechanics worsened at PEEP below AOP compared to those at PEEP levels above AOP, including lower respiratory system compliance (9 ± 2 mL/cmH_2_O vs. 13 ± 2 mL/cmH_2_O, *p* = 0.003), higher transpulmonary driving pressure (26 ± 3 cmH_2_O vs. 19 ± 4 cmH_2_O, *p* = 0.007) (Table E1), and lower percentage of dorsal ventilation (Figure E4). In bilateral lung injury, when exposed to PEEP below AOP, both right and left lungs showed significantly lower compliance than at PEEP above AOP. The same was true for the dorsal compliance but not for the ventral one (Table E1). In the case of asymmetrical injury, increasing PEEP above AOP did not change regional compliance. Compared to PEEP above AOP, delta end-expiratory lung volume was lower at low PEEP in both groups (Figure E4). However, the effects of iNO on end-expiratory lung impedance were not significantly affected by PEEP (Table E1). The median values of selected PEEP above AOP were 3cmH_2_O (Min:2cmH_2_O - Max:12cmH_2_O), and 0 cmH_2_O (Min:0cmH_2_O - Max:3cmH_2_O) below AOP, for asymmetrical injury. There were no differences in respiratory mechanics between PEEP below versus above AOP in the asymmetrical group.

### Pulmonary and systemic hemodynamics

**Fig. 4:**
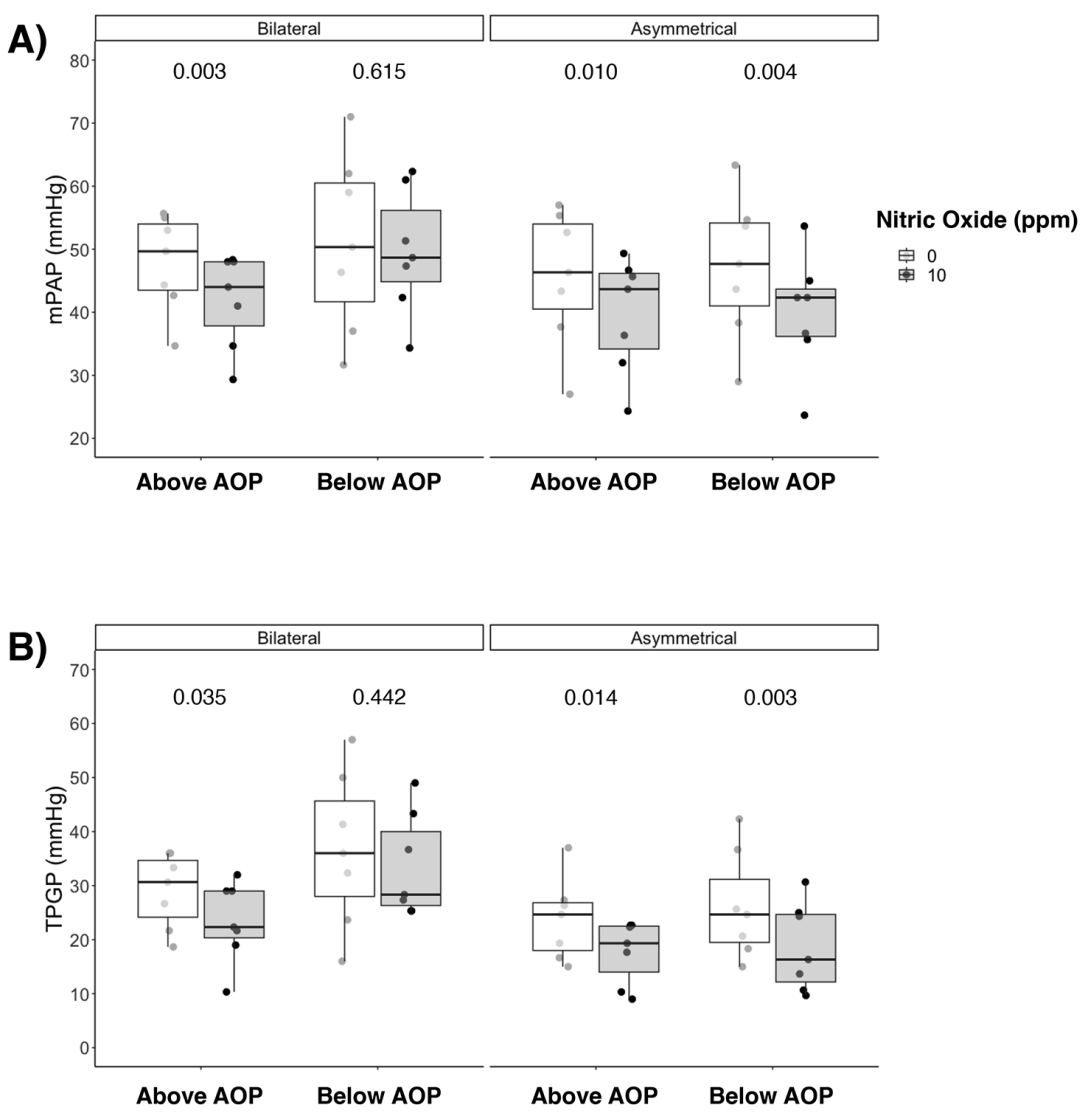
Effects of iNO on pulmonary hemodynamics. **(A)** Mean Pulmonary Artery Pressure (mPAP) and **(B)** Transpulmonary Gradient Pressure (TPG) response to inhaled nitric oxide according to Positive End-Expiratory Pressure (PEEP), i.e., below Airway Opening Pressure (AOP) or above AOP, and type of lung injury, i.e., bilateral or asymmetrical. Note: p-values are from univariate analysis (paired t-test or Wilcoxon test)

In the bilateral injury group, iNO decreased mPAP when PEEP was set above AOP (49 ± 8 to 42 ± 7mmHg; *p* = 0.003), but not when PEEP was set below AOP (51 ± 14 to 50 ± 10mmHg; *p* = 0.615) (Fig. 4A). PVR presented a behavior similar to mPAP. However, in the bilateral group at PEEP above AOP the decrease in PVR was not statistically significant, *p* = 0.067 (Figure E5). The heart rate decreased with iNO at both levels of PEEP (PEEP below AOP:181 ± 38 vs.163 ± 42 bpm, *p* = 0.007; PEEP above AOP: 136 ± 27 vs. 125 ± 24 bpm, *p* = 0.013), and cardiac output was higher without iNO at PEEP below AOP (8.1 ± 2.2 vs. 7.2 ± 2.2 L/min, *p* = 0.048) in the bilateral group. In the asymmetrical group, iNO was associated with reduction of mPAP with PEEP set both below (47 ± 11 to 40 ± 9mmHg; *p* = 0.004) and above AOP (46 ± 11 to 40 ± 9mmHg; *p* = 0.010). There were no differences in systemic hemodynamics in the asymmetrical group (Table E2). Multivariate analysis showed an effect of iNO (*p* = 0.001) and of PEEP (*p* = 0.039) on mPAP and interaction between iNO, PEEP and lung injury group (*p* = 0.036). The transpulmonary vascular-pressure gradient followed the same behavior as mPAP, with effect of PEEP (*p* = 0.010) and iNO (*p* = 0.002) (Fig. 4B).

### Gas exchange and regional ventilation/perfusion distribution

**Fig. 5:**
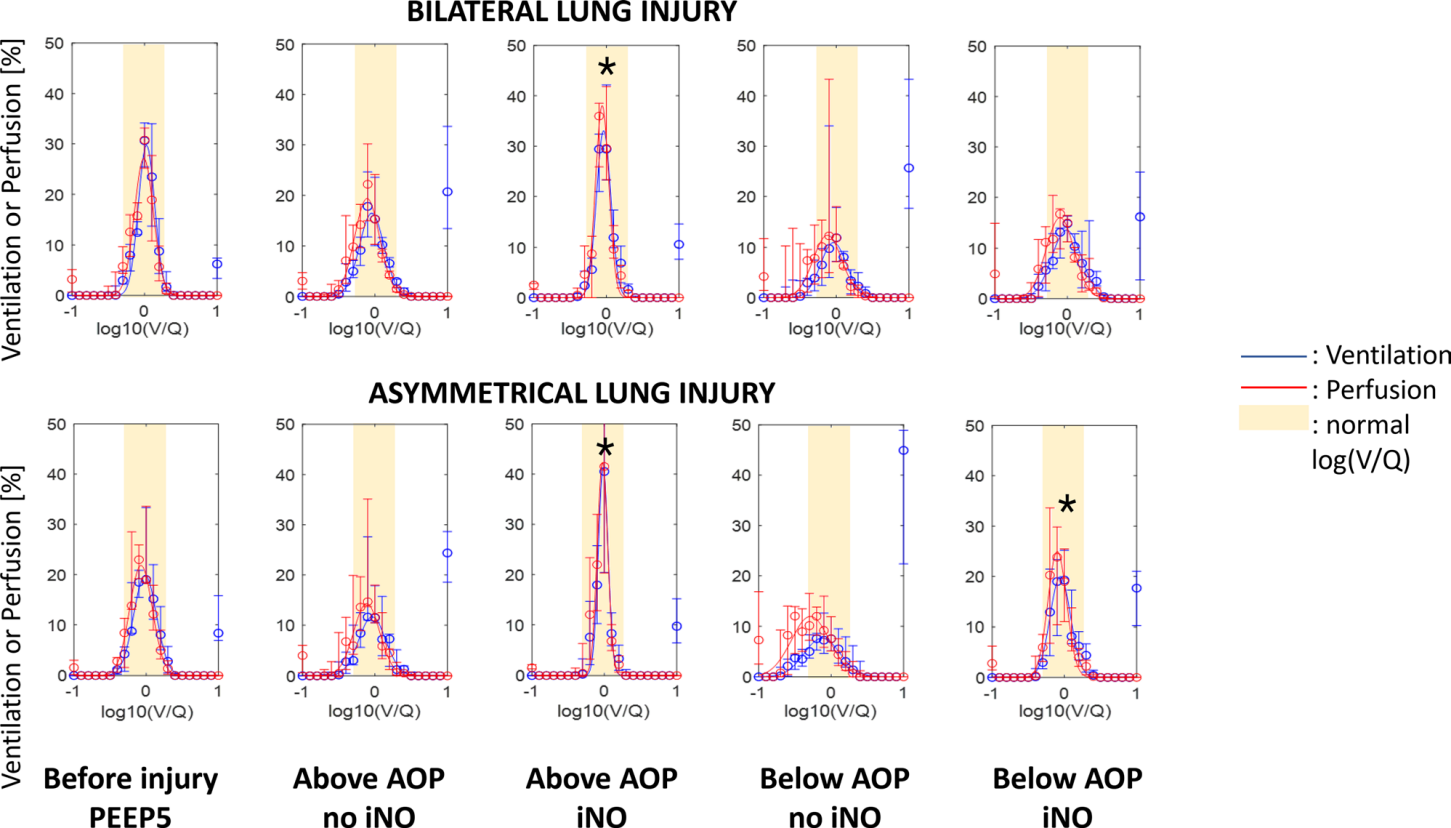
Ventilation/perfusion analysis based on electrical impedance tomography. **x-axis**: log10(V/Q) where 0 indicates ventilation equal to perfusion; -1 indicates perfusion 10 times higher than ventilation, and + 1 indicates ventilation 10 times higher than perfusion. Moreover, the variable on the x-axis is analysed and reported as discrete, where all original values are approximated to the closest fist decimal number between − 1 and + 1, originating 21 possible values. Log(V/Q) = -1 reports all log(V/Q) values equal or lower than − 1. Log(V/Q) = 1 reports all log(V/Q) values equal or higher than + 1. **y-axis**: regional distribution of the fraction of ventilation (blue) and perfusion (red), indicated as percentage of the total impedance in the corresponding (ventilation or perfusion) map. Values reported as median (IQR) and represented as by open circles and whiskers. The solid lines correspond to the best fitting curves (with 95%CI). *: to mark significant differences compare to the correspondig ventilatory condition without iNO (Two-sided Wilcoxon signed rank test; α = 0.05). The yellow area indicating normal V/Q between 0.5 and 2 corresponding to log(V/Q) between − 0.3 and 0.3. Abbreviations: V/Q: ventilation/perfusion ratio; PEEP: positive end-expiratory pressure; AOP: airway opening pressure; iNO: inhaled nitric oxide

**Fig. 6:**
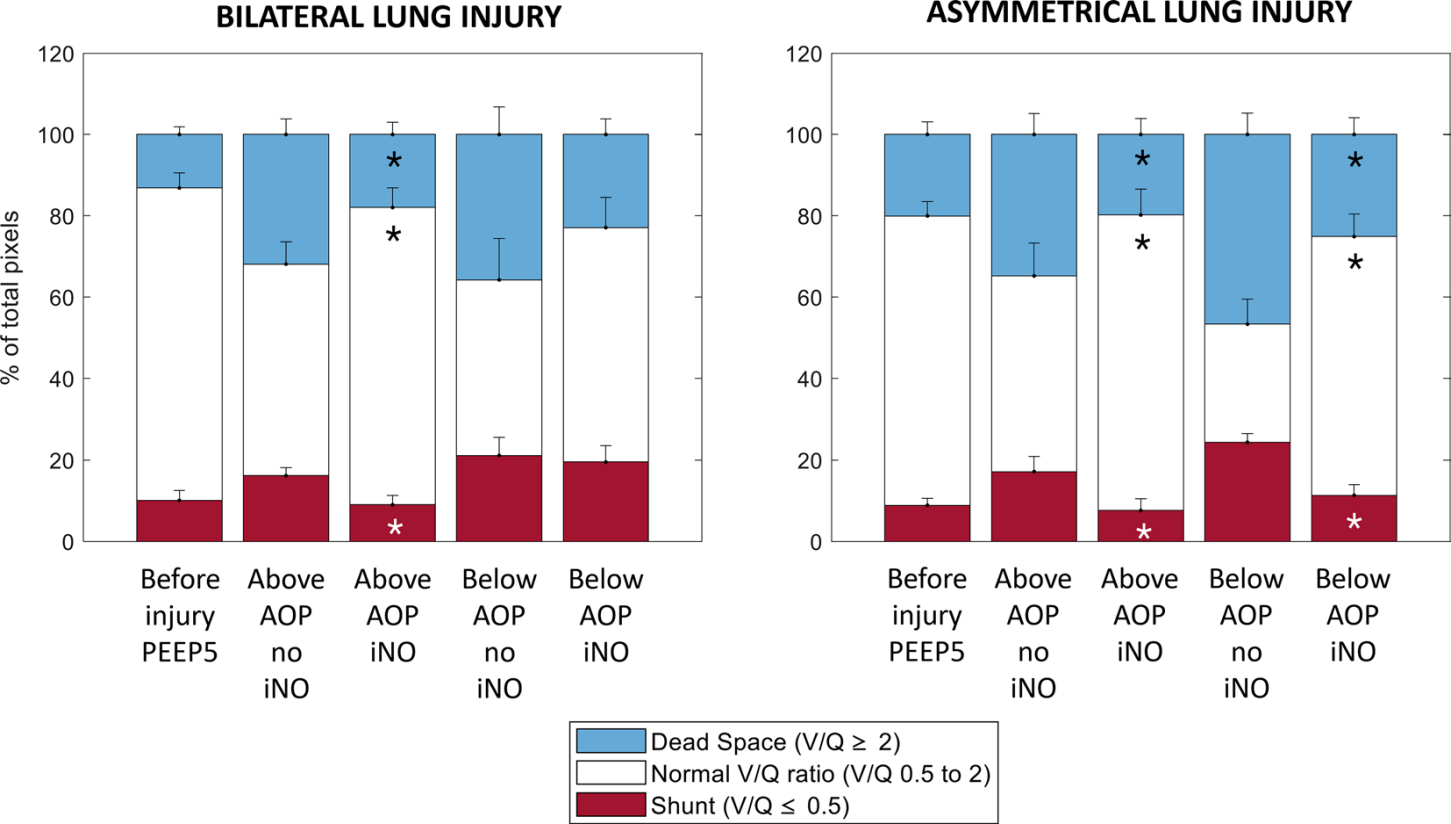
Percentages of pixels for each lung compartment: **(1) shunt (red)** defined as log(V/Q) lower than or equal to -0.3 or as V/Q lower than or equal to 0.5; **(2) normal V/Q (white)** defined as a V/Q between 0.5 and 2; **(3) dead space (blue)** defined as log(V/Q) equal to or higher than 0.3 or as V/Q equal to or higher than 2. Values reported as mean + SEM. Abbreviations: V/Q: ventilation/perfusion ratio; PEEP: positive end-expirtory pressure; AOP: airway opening pressure; iNO: inhaled nitric oxide. ). *: to mark significant differences compare to the correspondig ventilatory condition without iNO (Two-sided Wilcoxon signed rank test; α = 0.05)

Gas exchange response to iNO is reported in Table 2. Compared to ventilation without iNO, PaO_2_/FIO_2_ was higher under iNO in all studied conditions. PaCO_2_ decreased with iNO at both PEEP levels in the bilateral group and at PEEP below AOP in the asymmetrical group. The analysis of EIT-derived ventilation and perfusion maps (one pair of maps for each studied condition) showed that the exposure to iNO significantly modified the V/Q ratio: both peak and kurtosis of the V/Q curves increased significantly when pigs were exposed to iNO therapy (Table E3). This response occurred only for PEEP above AOP in bilateral lung injury but independently from the selected PEEP in asymmetrical lung injury (Fig. 5 and Table E3). The same results were confirmed when the lung was divided into three V/Q compartments: significant changes in the low V/Q and shunt compartment, the high V/Q and dead space compartment, and the normal V/Q were observed. In case of bilateral lung injury, the effects of iNO on V/Q were only visible at PEEP above AOP, but hampered when PEEP was set below AOP (Fig. 6 and Tables E4 and E5). When pigs with asymmetrical lung injury were exposed to iNO, independently from PEEP levels, iNO improved normal V/Q, reducing areas of dead space and shunt. The multivariate analysis showed an effect of iNO on both reduction of low V/Q and dead space and of increase of normal V/Q (*p* < 0.001). PEEP (above vs. below AOP) and distribution of lung injury (bilateral vs. asymmetrical) showed only an effect on normal V/Q (*p* = 0.01).

**Table 2:** Gas exchange response to inhaled nitric oxide (iNO)

Variable	Asymmetrical Lung Injury (*n* = 7)								Bilateral Lung Injury (*n* = 7)					
	Below AOP			Above AOP					Below AOP			Above AOP		
	zero iNO	10 ppm iNO	*p*	zero iNO	10 ppm iNO	*p*			zero iNO	10 ppm iNO	*p*	zero iNO	10 ppm iNO	*p*
PaO_2_/FiO_2_, mmHg	125 ± 82	197 ± 107	0.010	158 ± 87	275 ± 118	0.004			50 ± 13	65 ± 20	0.012	97 ± 25	164 ± 75	0.027
ETCO_2_, mmHg	45 ± 10	47 ± 10	0.186	49 ± 10	52 ± 8	0.181			50 ± 10	51 ± 12	0.812	52 ± 8	55 ± 8	0.303
PaCO_2_, mmHg	66 ± 17	59 ± 18	0.012	63 ± 13	61 ± 16	0.117			84 ± 17	77 ± 14	0.015	71 ± 17	68 ± 14	0.045
Vd/Vt	0.31 ± 0.10	0.18 ± 0.14	0.004	0.21 ± 0.10	0.12 ± 0.16	0.090			0.39 ± 0.10	0.34 ± 0.08	0.273	0.24 ± 0.19	0.18 ± 0.09	0.230
Arterial pH	7.26 ± 0.09	7.30 ± 0.12	0.006	7.27 ± 0.08	7.30 ± 0.09	< 0.001			7.19 ± 0.10	7.22 ± 0.09	0.008	7.24 ± 0.10	7.26 ± 0.10	0.672
BE, mmol/L	-0.3 ± 8.2	1.9 ± 2.9	0.529	2.0 ± 2.5	2.0 ± 2.9	0.984			2.8 ± 2.3	3.2 ± 2.6	0.402	2.1 ± 2.7	3.4 ± 2.3	0.042

*Abbreviations* PEEP, positive end-expiratory pressure; AOP, airway opening pressure; iNO, inhaled nitric oxide; PaO_2_/FiO_2_, arterial oxygen partial pressure and fraction of inspired oxygen ratio; ETCO_2_, end-tidal carbon dioxide; PaCO_2_, arterial carbon dioxide partial pressure; Vd/Vt, dead space and tidal volume ratio, Bohr-Enghoff equation; BE, base excess. *Notes*: Data are presented as mean and standard deviation. p-values are from univariate analysis (Paired t-test or Wilcoxon test)

## Discussion

This randomized experimental study describes the impact of distribution of lung injury and PEEP setting in relation to AOP, on the efficacy of iNO therapy in ARDS in terms of gas exchange and hemodynamics. Our main findings can be summarized as follows:

- In bilateral injury, the effects of iNO on pulmonary hemodynamics, including reduction of mPAP, were observed only when PEEP was set above AOP, while in asymmetrical lung injury, iNO improved pulmonary hemodynamics regardless of the set PEEP.
- iNO therapy improved oxygenation regardless of PEEP set and distribution of lung injury.
- In bilateral lung injury, iNO improved regional V/Q match, reducing dead space and shunt for the benefit of larger areas with normal V/Q, only when PEEP was set above AOP. This effect existed regardless of PEEP in asymmetrical lung injury.
- The beneficial effects of iNO on pulmonary hemodynamics and V/Q distribution was therefore very strongly influenced by the selected PEEP in relation to AOP in bilateral lung injury. Bilateral lung injury and PEEP level below AOP hinder iNO beneficial hemodynamic effects and illustrate the possible reduced efficacy of iNO or any other inhaled therapy in clinical practice if airway closure is not taken into account. One can speculate that the difference in iNO efficacy between bilateral and asymmetrical injury is attributable to the more prolonged patency of the airways characterising the less injured lung in the asymmetrical injury.

### Airway closure and the asymmetrical model

Our model of lung injury resulted in airway closure in all animals, similar to the prevalence of 90% reported by Bastia et al. [27] using the same models of lung injury in pigs when we defined the presence of AOP as any level above 0.5 cmH_2_O. The pathophysiology of airway closure is not completely understood: some of the mechanisms proposed, which are also present in our model, are reduced functional residual capacity, surfactant depletion, and high chest wall elastance [35]. The combination of these factors may increase the risk of airway closure during expiration due to changes in the balance of forces acting on the airways and lung tissue [35, 36]. As expected, despite physiological variability leading to an inhomogeneous distribution of injury between the two lungs, AOP did not differ between the left and right lungs in the case of bilateral lung injury. For asymmetrical injury, despite the AOP tending to be slightly higher in the lung that was submitted to lavage and high stretch ventilation (left lung), AOP was not statistically different between left and right lung. As the right lung was not ventilated during the injury, but maintained collapsed for around 1 h, both lungs ended up injured by different mechanisms, therefore, even though the wet-to-dry ratios were different between right and left lungs in the dependent regions of asymmetrical injury, both lungs presented high values, suggesting a diffuse although asymmetrical injury.

### Respiratory mechanics, lung volume and regional distribution of ventilation

As expected, given the onset of airway closure and lung collapse, the global respiratory mechanics as well as the dorsal ventilation were negatively affected by PEEP levels below AOP in case of bilateral lung injury. Interestingly, this was much less pronounced in case of asymmetrical lung injury, where the healthier lung ensured an unchanged global respiratory mechanics (e.g., both global and regional respiratory system static compliance) and regional distribution of ventilation at the two tested PEEP values, confirming previous results [27]. Although a PEEP below AOP was associated with a significant reduction in end-expiratory lung volume, the influence of iNO on end-expiratory lung impedance was not significantly affected by PEEP.

### Hemodynamics and gas exchange

Our data indicate that the administration of iNO determines a redistribution of blood perfusion within the lungs, improving the pulmonary circulation and reducing mPAP and PRV, which presented similar behaviour without affecting systemic hemodynamics, consistent with other studies [37, 38] and representing one of the advantages of using the inhaled form of nitric oxide in clinical practice. As both PEEP and iNO can affect pulmonary hemodynamics, we performed a multivariate analysis that showed that iNO and PEEP can separately affect mPAP and transpulmonary pressure gradient, with an interaction between the effect of iNO, PEEP and the distribution of lung injury on mPAP and PVR.

In our study, iNO therapy improved oxygenation regardless of PEEP level or distribution of lung injury, which is in line with a broad literature [20], confirming that improved blood oxygenation may not be necessarily correlate with improved pulmonary hemodynamics, better V/Q matching, and better outcomes. Interestingly, a significant reduction of PaCO_2_ was observed during iNO administration. As no ventilatory changes were performed while administering iNO, and minute ventilation was kept constant during each study phase, two possible explanations for the improved CO_2_ clearance coexist: (1) a significant reduction in dead space during iNO administration which is likely to occur with iNO, and (2) the Haldane effect, a property of the hemoglobin which enhances the displacement of carbon dioxide due to improved oxygenation in the pulmonary circulation, increasing CO_2_ clearance [39]. The significant decrease in heart rate during iNO exposure, which typically has minimal or no effect on systemic hemodynamics, can be explained by improved oxygenation, acidosis or PVR, all leading to a reduction in sympathetic drive.

### Regional ventilation/perfusion distribution

Despite its clinical relevance, the regional distribution of V/Q is difficult to assess at the bedside and is rarely monitored in the intensive care. Several techniques have been proposed, all having limitations in the ICU [40, 41]. Dual-energy computed tomography is a recent imaging technique that has provided valuable information about the influence of therapies and ventilator settings on regional V/Q, with high spatial resolution [42–44]. However, the technique needs intrahospital transport of critically ill patients and exposure to ionizing radiation. Although with low spatial resolution, EIT is a radiation-free non-invasive imaging modality that allows to continuously monitor the distribution of ventilation and V/Q at the bedside [45]. A few studies have recently shown that unmatched ventilation and perfusion estimated by EIT predicts outcomes in ARDS patients [32, 46, 47]. To our knowledge, only one study has previously investigated the effects of iNO on regional perfusion [37], unveiling a variety of patterns of perfusion changes in response to iNO. Based on our results, PEEP level needs to be higher than the highest regional AOP to ensure an optimal efficacy of iNO in bilateral lung injury. As expected, given its mechanics of action, iNO mostly reduced total dead space and low V/Q and consequently expanded the areas with normal V/Q. This was quantitatively confirmed by a significant increase in kurtosis and peak of both the ventilation and perfusion curve when iNO was administered while PEEP was set above AOP. At PEEP below AOP, iNO modified ventilation and perfusion only if the lung injury was asymmetrical. Although having a significant influence on iNO effects on the overall distribution of V/Q and normal V/Q, the type of injury (bilateral vs. asymmetrical) was not associated with high V/Q and dead space. The persistent effect of iNO on mPAP and V/Q ratio at low PEEP in the asymmetrical group, as well as the improvement in oxygenation regardless PEEP, can likely be attributed to two factors: (1) the inhomogeneous distribution of injury with one lung relatively uninjured and, as such, open throughout the whole breathing cycle; (2) tidal recruitment, consequent to a tidal end-expiratory collapse related to AOP. Hence, we cannot exclude the possibility that iNO may be trapped in the alveoli during expiration, which might play a role in our results. In this regard, it should be considered that setting PEEP below AOP does not exclude a tidal opening of airways when the inspiratory pressure is above AOP. On the other hand, the onset of airway closure will prematurely interrupt expiration and, consequently, affect gas rheology on distal airways.

### Clinical implications

Our results may have important implications regarding inhaled therapy during mechanical ventilation [48], particularly for the use of iNO in ARDS as rescue therapy. This is the first study to show that the effect of iNO can be influenced by the applied PEEP in relation to regional airway closure. We also showed that the distribution of lung injury and lung recruitability may play a role in the iNO response. This observation provides one possible explanation for the discordance between the strong pathophysiological effects of iNO in ARDS and the lack of evidence of iNO efficacy improving patients’ outcomes.

### Limitations

In several animals, we used lower PEEP values and iNO doses than frequently used in clinical settings. The use of PEEP values lower than the AOP was needed to pursue the aims of this study. A relatively low dose of iNO was needed to damper its selective pulmonary vasodilator effects and reveal changes in iNO delivery consequent to the onset of airway closure. The study results can be specific to the selected injury model (lung lavage and injurious ventilation), potentially limiting generalizability [49, 50]. This is inevitable for translational research, but, at the same time, it isolates pathophysiological mechanisms that are impossible to investigate in clinical studies. AOP levels were low in some animals, which would have prevented the setting of PEEP below in a clinical scenario. Therefore, results are not all directly generalisable to a clinical context, but this experimental study demonstrates important pathophysiological mechanisms. However, in several animals this corresponded to relatively high PEEP settings (15 cmH_2_O) and the effect was consistent across all ranges of values. This study is hypothesis-generating and requires further validation in clinical settings.

For reasons related to the EIT image analysis which may ignore some regions never ventilated, the shunt estimated by EIT may be underestimated compared to a classical technique like the Multiple Inter Gas Elimination Technique [40]. Our findings are in line with previous results [32, 47] and are related to the definition of non-ventilated and non-perfused pixels. A change of the thresholds used in favor of perfusion (with a lower threshold to define perfused areas, compared to the threshold to define ventilated areas) would result in an increase in shunt. Finally, the design of the study and the EIT imaging does not allow discerning the effects of airway closure alone and/or the combination with lung collapse especially at very low PEEP.

## Conclusion

The distribution of lung injury (bilateral versus asymmetrical), the consequent lung collapse, and the PEEP level in relation to regional AOP are factors that influenced the efficacy of an inhaled therapy. This could be further investigated in clinical settings for any inhaled therapy. In a swine model of severe ARDS, we demonstrated that iNO is only effective at PEEP above AOP in bilateral lung injury, in terms of pulmonary hemodynamics and V/Q distribution. These findings have potential clinical implications, demonstrating that both distribution of lung injury and PEEP setting can affect iNO efficacy and the need to assess the presence of airway closure and AOP.

## Electronic supplementary material

Below is the link to the electronic supplementary material.

Supplementary Material 1: Table E1. Respiratory mechanics response to PEEP . Table E2. Systemic hemodynamics during inhaled Nitric Oxide (iNO) therapy. Table E3. Kurtosis and peak values obtained from the ventilation/perfusion analysis based on electrical impedance tomography. Table E4. Lung compartments analysis for each studied lung condition. Percentage of pixels. (compared to sum of all ventilated and/or perfused pixels) belonging to each lung compartment and lung condition. Table E5. Lung compartments analysis for each studied lung condition. Absolute number of pixels for each lung compartment and lung condition.

Supplementary Material 2: Figure E1. Representative case of lung ventilation monitored by Electrical Impedance Tomography (EIT) in the Asymmetrical Group. (A) ventilation of both lungs with single-lumen tube before lung injury; (B) ventilation of the left lung only, with double-lumen tube, after 2-hit lung injury. In the images ventilated areas are presented in blue-to-white scale and non-ventilated areas in black. Figure E2. A representative case of low flow PV curves and airway opening pressure (AOP) defection. PV curves performed after lung injury. Paw on the x-axis, volume [ml] estimated based on the correspondence between inspiratory impedance changes and inspiratory volume at the end of the PV curve maneuver. Data tips indicate the Paw corresponding to AOP for 1) the global respiratory system (right + left lung) - plot above; 2) the right lung and 3) the left lung – plots below. Figure E3. Lung wet-to-dry ratio. A) Non-dependent lung regions. B) Dependent lung regions. p-values are from univariate analysis (t-test or Mann-Whitney test, according to distribution). Figure E3. Lung wet-to-dry ratio. A) Non-dependent lung regions. B) Dependent lung regions. p-values are from univariate analysis (t-test or Mann-Whitney test, according to distribution). Figure E4. Changes in (A) end-expiratory lung volume (?EELV) and (B) dorsal ventilation consequent to changes in positive end-expiratory pressure (PEEP). PEEP above airway opening pressure (AOP) reported in white versus PEEP below AOP reported in gray.

## Data Availability

Data are available from the corresponding author on reasonable request.

## Abbreviations

AOP: Airway Opening Pressure
ARDS: Acute Respiratory Distress Syndrome
C_RS_: Respiratory System Static Compliance
EIT: Electrical Impedance Tomography
F_I_O_2_: Inspired Oxygen Fraction
iNO: inhaled Nitric Oxide
IQR: Interquartile Range
mPAP: mean Pulmonary Artery Pressure
NaCl: Sodium Chloride
PaCO_2_: Pressure of Carbon Dioxide in Arterial Blood
PaO_2_: Pressure of Oxygen in Arterial Blood
PAP: Pulmonary Artery Pressure
PVR: Pulmonary Vascular Resistance
PEEP: Positive End-Expiratory Pressure
R/I: Recruitment-to-Inflation Ratio
V/Q: Ventilation/Perfusion Ratio
