# Supplementary material for: Impact of airway closure and lung collapse on inhaled nitric oxide effect in acute lung injury: an experimental study
Source: Ann Intensive Care. 2024 Sep 23;14:149. doi: 10.1186/s13613-024-01378-z (PMC11420414; doi:10.1186/s13613-024-01378-z)
Supplement: Supplementary file 1 — Supplementary Material 1: Table E1. Respiratory mechanics response to PEEP . Table E2. Systemic hemodynamics during inhaled Nitric Oxide (iNO) therapy. Table E3. Kurtosis and peak values obtained from the ventilation/perfusion analysis based on electrical impedance tomography. Table E4. Lung compartments analysis for each studied lung condition. Percentage of pixels. (compared to sum of all ventilated and/or perfused pixels) belonging to each lung compartment and lung condition. Table E5. Lung compartments analysis for each studied lung condition. Absolute number of pixels for each lung compartment and lung condition. [file 13613_2024_1378_MOESM1_ESM.docx]

**Online Data Supplement**

Impact of airway closure and lung collapse on inhaled Nitric Oxide effect in acute lung injury: An experimental study

Mariangela Pellegrini*; Mayson L. de A. Sousa*; Sebastian Dubo; Luca S. Menga; Vanessa Hsing; Martin Post; Laurent J. Brochard

*Shared first authorship

**Extended Methods**

This was a randomized experimental study in two porcine models of ARDS, respectively with bilateral and asymmetrical injury. The experiments followed the Canadian Animal Care guidelines, and the protocol was approved by the local Animal Care Committee (Reference number: AUP 58058) of the Peter Gilgan Research and Learning Centre from The Hospital for Sick Children, Toronto, Canada.

*Animal Sample and Experiment Series*

The animal sample size was calculated based on a previous study assessing the effect of iNO on pulmonary artery pressure pre and post iNO (drop from 23±1.8 to 19±1 mmHg). Assuming a power of 0.8 and α-level of 0.05 a minimal sample size of five animals per group would be necessary. In addition to our calculated sample size, we performed four extra experiments (two animals per group) aiming to achieve relatively broad levels of AOP and to support the translation of our findings, resulting in a total sample of 14 animals (seven animals per group). The only difference in the second series of experiments (4 extra experiments mentioned before) was that we placed an abdominal binder and a 5-kg sandbag on the belly of the animal during the entire experiment. Our hypothesis was that these interventions would change the mechanics of the chest wall promoting airway closure and the level of AOP after lung injury. In all animals included in this study we followed the same protocol for animal preparation, lung injury, iNO and PEEP interventions, and data collection.

*Animal Preparation*

All pigs were initially sedated with intramuscular ketamine (15 mg/kg) and isoflurane (3%) and then orotracheally intubated (orotracheal tube diameter of 8.0 mm). After intubation, an infusion of pentobarbital at 5-10 mg/kg/h was started. When the pig no longer had a pain response to a hard toe pinch, a constant infusion of rocuronium (0.2mg/kg/h) for muscle paralysis was started. Intravenous infusion of Ringer’s Lactate (5-10 ml/kg/h) was used for fluid therapy throughout the whole experiment. During animal preparation, pigs were supine and mechanically ventilated with a baseline ventilation set as volume controlled ventilation, inspiratory fraction of oxygen (FIO_2_) of 50%, tidal volume (Vt) of 8ml/kg, positive end-expiratory pressure (PEEP) of 5 cmH_2_O, respiratory rate (RR) of 30 breaths per minute (bpm) and inspiratory-expiratory ratio (I:E) of 1:2. A nasogastric catheter equipped with an esophageal balloon (Nutrivent^®^, Mirandola, Italy) was inserted and calibrated following standard procedures [1]. The occlusion test was performed three times to reach an airway pressure to esophageal pressure ratio between 0.8 and 1.2, was done to confirm the correct catheter position[2]. For hemodynamics monitoring and blood sampling, the left femoral artery was cannulated with a PiCCO catheter (Getinge, Solna, Sweden) and the right external jugular vein was cannulated with an introducer for the pulmonary artery catheter (Swan-Ganz pulmonary artery catheter, Edwards Lifesciences, Irvine, United States). An electrical impedance tomography (EIT) belt (Pulmovista 500, Dräger, Lübeck, Germany), was placed around the thorax, just under the front legs of the animal. Peripheral oxygen saturation was monitored throughout the whole experiment (Siemens SC 9000XL Monitor, Siemens Healthineers, Erlangen, Germany). Baseline measurements were performed after a progressive and monitored recruitment maneuver characterized by pressure control ventilation, driving pressure of 15 cmH_2_O, RR of 30 bpm, I:E ratio of 1:1, and a stepwise increase in PEEP from 15 to 30 cmH_2_O.

*Lung Injury*

Lung injury was induced by a 2-hit model: a first hit characterized by surfactant depletion obtained by repeated lung lavages with 30ml/kg of saline at 37ºC until arterial oxygen pressure (PaO_2_) was stable below 100 mmHg with a FIO_2_ of 1, followed by a second hit composed by high stretch ventilation obtained by setting pressure controlled ventilation at the lowest PEEP possible to keep peripheral oxygen saturation (SpO_2_) equal to or higher than 90% and ranging between 1 and 13 cmH_2_O and a peak pressure of 40 cmH_2_O for 30 minutes. Pigs were randomized between two groups: bilateral lung injury group, in which the 2-hit lung injury was performed in both lungs, and asymmetrical lung injury group, in which the 2-hit lung injury was performed only in the left lung after a selected lung intubation. We used Block Randomization. We had two blocks of experiments first 10 experiments (5/ groups) and a second block of 4 experiments (2/group). In each block we use the drawing lots method. In the asymmetrical lung injury group, after baseline measurements (before injury), the single-lumen endotracheal tube was replaced with a double-lumen endotracheal tube and ventilation was maintained only in the left lung, while the right lung collapsed. Single lung ventilation was confirmed by EIT (Figure E1). After the 2-hit injury, ventilation of both lungs was restored, changing back to the single-lumen endotracheal tube. In the bilateral lung injury group, the single-lumen tube was maintained during the entire protocol. Lung injury measurements were performed after lung injury (with single-lumen tube) in all animals. Low-flow inflation maneuvers (5 L/min) were performed at the baseline, after lung injury, and at the end of each PEEP step, to measure global and regional AOP by EIT. The AOP measurements after lung injury were used to guide the following interventions. The exact AOP value detection was performed offline by plotting the respiratory system pressure-volume curve collected in quasi-static conditions and the respiratory circuit pressure-volume curve, which was measured before the start of the experiment. The AOP was defined as the point of abrupt increase in compliance above the respiratory circuit compliance.

We also identified global and regional (for each lung) AOP based on EIT. As EIT measures lung aeration at the regions where the EIT belt is placed, very small increase in aeration suggests that the airways are open, as observed in previous publication [3]. In our study we defined AOP as the level of pressure to which the estimated delta volume (estimated by EIT) was higher than 0.2 ml.

*Interventions (iNO and PEEP)*

All animals were submitted to low-tidal volume ventilation (6-8 mL/kg) with two levels of PEEP: 1) PEEP above AOP, set 2 cmH_2_O above the highest regional AOP (Figure E2); 2) PEEP below AOP, set below the global or the lowest regional AOP. At each level of PEEP, the pigs were ventilated for 10 min without iNO and for 10 min with iNO at 10 ppm (Air Liquide S.A. Paris, France) administered by SoKINOX (INOsystems, Air Liquide Healthcare, Paris, France). There was a “washout period” of 10 additional minutes between each step. In each experiment, we randomized the order of PEEP (below AOP vs above AOP) and the order of iNO administration (with vs without). As for radomization for asymmetrical and bilateral lung injury, we use the drawing lots method also for the order of PEEP and of iNO. Measurements were performed at the end of each 10 min step. An overview of the study protocol is presented in Figure 1. At the end of the experiment, the animals were euthanized with an overdose of pentobarbital. Samples of the dependent and non-dependent regions of each lung were collected for wet-to-dry ratio measurements.

*Data collection*

Pulmonary and systemic hemodynamics, gas exchange and respiratory mechanics were measured at baseline and, after lung injury, at the end of each studied condition. Pulmonary artery pressure, central venous pressure, systemic arterial pressure, heart rate, airway pressure, airway flow, and esophageal pressure were acquired at 1 kHz in LabChart (ADInstruments, Sydney, Australia). Airway flow and pressure were recorded at the airway opening through a heated-type pneumotachograph (Respiratory Flow Head 300L, ADInstruments), and tidal volume was calculated as the integral of the airway flow. Cardiac output, extra-vascular lung water (EVLW), and global end-diastolic volume (GEDV) were estimated by Pulsion PiCCO (Getinge, Solna, Sweden), as an average of three measurements for each time point. Mixed venous and arterial blood samples were analyzed by Stat Profile Prime Plus VET blood gas analyzer (Nova Biomedical, Massachusetts, USA). Synchronized ventilator (Evita Infinity V500, Dräger, Lübeck, Germany) and EIT tracings (Pulmovista 500, Dräger, Lübeck, Germany) were continuously recorded with a sample rate of 50 Hz. For the acquisition of ventilation and perfusion EIT maps, a 20-seconds inspiratory-hold at mean airway pressure was performed. During the hold, a 10 ml bolus of a hypertonic natrium chloride (5% NaCl) solution was rapidly infused through the central line to assess lung perfusion. EIT data were subsequently processed offline by commercial software (EIT Perfusion Analysis SW Version 1.2.0, Dräger) to obtain 32x32 ventilation and perfusion EIT maps (Figure E3).

*Data analysis*

Mean pulmonary artery pressure (mPAP) was calculated as (2/3 of pulmonary artery diastolic pressure) + (1/3 of pulmonary artery systolic pressure). Mean arterial pressure (MAP) was calculated as (2/3 of diastolic arterial pressure) + (1/3 of systolic arterial pressure). Transpulmonary vascular-pressure gradient (TPG) was calculated as mPAP minus wedge pressure, and pulmonary vascular resistance (PVR) was calculated as TPG divided by cardiac output (CO). End-expiratory lung impedance (EELI) was measured using an EIT dedicated software for offline analysis (PV500 Analysis, Dräger.) All EIT files were combined and EELI was computed as an averaged of EELI from 5 breaths in each time point. Regional respiratory system compliance (C_RS_) was estimated as total C_RS_ measured by the ventilator times percentage of aeration in the specific regions of interest. EIT-derived ventilation and perfusion maps were analyzed at a pixel level using custom-made scripts developed by one of the authors (MP) using MatLab programming (MATLAB R2023b, MathWorks, Natick, MA, USA). Based on previous literature, each pixel unit of the ventilation map was considered not ventilated if its corresponding impedance value was below 20% of the highest pixel-based impedance reached in the same map. The same threshold was applied for the prefusion map. The V/Q analysis was conducted following the method previously proposed by Pavlowsky et al. [4] and summarized as following. For both the ventilation and the perfusion map, each pixel impedance was expressed as percentage of the global impedance of the corresponding map. Subsequently, the log(V/Q) was calculated pixelwise. To plot the log(V/Q) distribution, log(V/Q) values were rounded to their closest first decimal. Log(V/Q) values below or equal -1 (corresponding to a V/Q lower or equal 0.1) were classified as complete shunt and rounded to -1, whereas log(V/Q) values equal or above 1 (corresponding to a V/Q equal or above 10) were classified as complete dead space and rounded to +1. The combination of discretization of V/Q values and aggregation of pixel classified as complete shunt and complete dead space, led to define 21 log(V/Q) compartments between -1 and 1, with increments of 0.1. The pixel-based log(V/Q) (x-axis), calculated as above, was then plotted against the mean pixel-based percentage of ventilation and of perfusion (y-axis). These distribution curves were then fitted with a Gaussian curve. The fitting model robustness was checked based on the residual sum of squares (RSS) analysis and excluding the two extreme log(V/Q) compartments equal to -1 and to 1. Peak value, and kurtosis of the Gaussian fitting curves, both for the ventilation and the prefusion curves, were used to quantify differences among distribution curves for all studied conditions. The total area under the curve for both ventilation and perfusion V/Q curve was also estimated, this to investigate significant changes in global ventilation and perfusion when the lung were exposed or not to iNO at the same PEEP level. Based on previously proposed classification of shunt and dead space [5,6], a three-compartment model was designed and applied to the log(V/Q) pixel values to define: 1) shunt with a V/Q ratio equal to or lower than 0.5 and a log(V/Q) equal to or lower than -0.3; 2) normal V/Q ratio corresponding to a V/Q ratio between 0.5 and 2 and a log(V/Q) between -0.3 and 0.3; 3) dead space with units with high V/Q ratio corresponding to a V/Q ratio equal to or higher than 2 and a log(V/Q) equal to or higher than 0.3.

*Animal Sample and Experiment Series*

In addition to our calculated sample size, we performed four extra experiments (two animals per group) aiming to achieve relatively broad levels of AOP and to support the translation of our findings, resulting in a total sample of 14 animals (seven animals per group). We run a post hoc power analysis considering the mean PAP with and without iNO at PEEP below AOP in the bilateral group sample and the post hoc power was 0.83.

*Statistical Analysis*

Categorical variables were presented as counts and proportions, and continuous variables were reported as mean and standard deviation (SD) or as median and interquartile range (IQR). [Multivariate Imputation by Chained Equations](https://search.r-project.org/CRAN/refmans/mice/help/mice.html) was applied to handle missing values. The presence of airway closure and the respective airway opening pressure (AOP) were calculated for each lung. The proportion of airway closure was reported overall and stratified by lung (right, left) and by injury (bilateral, asymmetrical). The proportions were compared between groups using Chi-square test or Fisher’s exact test for small samples. Univariate analyses, to assess the impact of iNO (comparisons of “with iNO” versus “without iNO”), were performed using paired Student’s t-test or Wilcoxon signed-rank test, for continuous variables, and Chi-square test or Fisher’s exact test, for categorical variables. Multivariate analyses were performed using Mixed Analysis of Variance (Mixed ANOVA) for variables with statistical significance in the univariate analysis. All tests were two-tailed with type-I error (alpha) set at 0.05. The statistical analyses were performed using MatLab (MATLAB R2023b, MathWorks, Natick, MA, USA) and the R software (<https://www.R-project.org/>).

**Results**

There was no missing data for our main outcome variable, pulmonary artery pressure. There was also no missing data for the oesophagal pressure and EIT variables. We had missing data for the PiCCO measurements at only one point, which did not affect the study's main results.

**References**

1. Jonkman AH, Telias I, Spinelli E, Akoumianaki E, Piquilloud L. The oesophageal balloon for respiratory monitoring in ventilated patients: updated clinical review and practical aspects. European Respiratory Review [Internet]. 2023 [cited 2024 Jan 6];32. Available from: https://err.ersjournals.com/content/32/168/220186

2. Baydur A, Behrakis PK, Zin WA, Jaeger M, Milic-Emili J. A simple method for assessing the validity of the esophageal balloon technique. Am Rev Respir Dis. 1982;126:788–91.

3. Victorino JA, Borges JB, Okamoto VN, Matos GFJ, Tucci MR, Caramez MPR, et al. Imbalances in Regional Lung Ventilation. Am J Respir Crit Care Med. 2004;169:791–800.

4. Pavlovsky B, Pesenti A, Spinelli E, Scaramuzzo G, Marongiu I, Tagliabue P, et al. Effects of PEEP on regional ventilation-perfusion mismatch in the acute respiratory distress syndrome. Crit Care. 2022;26:211.

5. Wagner PD, Laravuso RB, Uhi RR, West JB. Continuous Distributions of Ventilation-Perfusion Ratios in Normal Subjects Breathing Air and 100% O_2_. J Clin Invest. 1974;54:54–68.

6. Petersson J, Glenny RW. Gas exchange and ventilation–perfusion relationships in the lung. European Respiratory Journal. 2014;44:1023–41.

**Figure legends**

**Figure E1. Representative case of lung ventilation monitored by Electrical Impedance Tomography (EIT) in the Asymmetrical Group.** (**A**) ventilation of both lungs with single-lumen tube before lung injury; (**B**) ventilation of the left lung only, with double-lumen tube, during 2-hit lung injury. Ventilated areas are presented in blue-to-white scale and non-ventilated areas in black.

**Figure E2. A representative case of low flow PV curves and airway opening pressure (AOP) defection.** PV curves performed after lung injury. Paw on the x-axis, volume [ml] estimated based on the correspondence between inspiratory impedance changes and inspiratory volume at the end of the PV curve maneuver. Data tips indicate the Paw corresponding to AOP for 1) the global respiratory system (right + left lung) - plot above; 2) the right lung and 3) the left lung – plots below.

**Figure E3. Lung wet-to-dry ratio. A)** Non-dependent lung regions. **B)** Dependent lung regions. p-values are from univariate analysis (t-test or Mann-Whitney test, according to distribution).

**Figure E4**. **Changes in (A) end-expiratory lung volume (ΔEELV) and (B) dorsal ventilation consequent to changes in positive end-expiratory pressure (PEEP).** PEEP above airway opening pressure (AOP) reported in white versus PEEP below AOP reported in gray.

**Table E1.** Respiratory mechanics response to PEEP

|  |  | Asymmetrical Lung Injury(n=7) | | |  | Bilateral Lung Injury (n=7) | | |
| --- | --- | --- | --- | --- | --- | --- | --- | --- |
|  |  | PEEP  below AOP | PEEP  above AOP | p |  | PEEP  below AOP | PEEP  above AOP | p |
| Global C_RS_, mL/cmH_2_O |  | 14 ± 5 | 15 ± 4 | 0.069 |  | 9 ± 2 | 13 ± 2 | 0.003 |
| Pplat, cmH_2_O |  | 27 ± 10 | 26 ± 9 | 0.902 |  | 37 ± 8 | 33 ± 9 | 0.096 |
| Airway ΔP, cmH_2_O |  | 25 ± 9 | 21 ± 6 | 0.052 |  | 33 ± 4 | 24 ± 5 | 0.004 |
| Δ P_L_, cmH_2_O |  | 16 ± 7 | 13 ± 4 | 0.127 |  | 26 ± 3 | 19 ± 4 | 0.007 |
| Expiratory P_L_, cmH_2_O |  | -3 ± 4 | -3 ± 2 | 0.845 |  | -3 ± 5 | -1 ± 5 | 0.230 |
| Δ EELI, AU^4^ |  | -1.94 ± 0.67 | -1.52 ± 0.41 | 0.127 |  | -2.18 ± 0.67 | -1.86 ± 0.54 | 0.040 |
| Right C_RS_, mL/cmH_2_O^†^ |  | 8.7 ± 3.7 | 9.5 ± 3.0 | 0.189 |  | 4.6 ± 0.7 | 6.5 ± 1.3 | 0.007 |
| Left C_RS_, mL/cmH_2_O |  | 5.2 ± 2.1 | 5.8 ± 2.0 | 0.141 |  | 4.2 ± 1.5 | 5.7 ± 1.2 | 0.016 |
| Ventral C_RS_, mL/cmH_2_O^†^ |  | 10.1 ± 3.8 | 10.4 ± 3.8 | 0.677 |  | 6.2 ± 1.6 | 7.2 ± 1.5 | 0.061 |
| Dorsal C_RS_, mL/cmH_2_O |  | 3.9 ± 0.9 | 5.1 ± 2.4 | 0.469 |  | 2.6 ± 1.1 | 5.2 ± 1.9 | 0.002 |

*Abbreviations:* PEEP, positive end-expiratory pressure; AOP, airway opening pressure; C_RS_, respiratory system compliance; Pplat, plateau pressure; P_AW_, airway pressure; P_L_, transpulmonary pressure; EELI, end-expiratory lung impedance; AU, arbitrary unit.

**Table E2.** Systemic hemodynamics during inhaled Nitric Oxide (iNO) therapy

| Variable | Asymmetrical (n=7) | | | | | | Bilateral (n=7) | | | | | |
| --- | --- | --- | --- | --- | --- | --- | --- | --- | --- | --- | --- | --- |
|  | Below AOP | | | Above AOP | | | Below AOP | | | Above AOP | | |
|  | iNO=0 | iNO=10 | p | iNO=0 | iNO=10 | p | iNO=0 | iNO=10 | p | iNO=0 | iNO=10 | p |
| Heart rate, bpm | 121±23 | 120±25 | 0.877 | 121±19 | 115±16 | 0.185 | 181±38 | 163±42 | 0.007 | 136±27 | 125±24 | 0.013 |
| MAP, mmHg | 103±18 | 107±22 | 0.309 | 110±19 | 117±22 | 0.217 | 101±35 | 113±13 | 0.336 | 116±18 | 117±19 | 0.839 |
| SBP, mmHg | 117±19 | 123±25 | 0.246 | 126±18 | 134±22 | 0.119 | 115±38 | 128±10 | 0.373 | 136±16 | 135±21 | 0.738 |
| DBP, mmHg | 95±18 | 99±21 | 0.3897 | 103±20 | 109±22 | 0.308 | 93±34 | 105±15 | 0.320 | 107±20 | 108±19 | 0.611 |
| CO, L/min | 4.9±1.3 | 5.1±1.3 | 0.095 | 4.7±1.6 | 4.8±1.3 | 0.764 | 8.1±2.2 | 7.2±2.2 | 0.048 | 5.7±0.8 | 5.9±1.1 | 0.673 |

*Abbreviations:* PEEP, positive end-expiratory pressure; AOP, airway opening pressure; iNO, inhaled nitric oxide; MAP, mean arterial pressure; SBP, systolic blood pressure; DBP, diastolic blood pressure; CO, cardiac output.

**Table E3.** Kurtosis and peak values obtained from the ventilation/perfusion analysis based on electrical impedance tomography.

|  |  | **Healthy Lung**  **(PEEP 5cmH_2_O)** |  | **PEEP above AOP** | | | | | |  | **PEEP below AOP** | | | | | |
| --- | --- | --- | --- | --- | --- | --- | --- | --- | --- | --- | --- | --- | --- | --- | --- | --- |
|  |  |  |  | **Without iNO** |  | **With iNO** |  | **Signed rank (without vs with iNO)** |  |  | **Without iNO** |  | **With iNO** |  | **Signed rank (without vs with iNO)** |  |
|  |  | Median (IQR) |  | Median (IQR) |  | Median  (IQR) |  | p |  |  | Median  (IQR) |  | Median  (IQR) |  | p |  |
| **Kurtosis** | | | | | | | | | | | | | | | | |
| *Bilateral lung injury* | | | | | | | | | | | | | | | | |
| Ventilation |  | 6.69  (5.55 - 7.11) |  | 4.77  (2.61 - 7.98) |  | 7.09  (6.26 - 7.29) |  | **0.04 *** |  |  | 2.04  (1.37 - 7.55) |  | 2.84  (2.39 - 3.12) |  | 0.44 |  |
| Perfusion |  | 5.39  (3.58 - 8.13) |  | 6.26  (3.35 - 9.42) |  | 7.37  (6.52 - 8.62) |  | **0.04 *** |  |  | 3.68  (2.70 - 7.69) |  | 3.45  (3.19 - 7.17) |  | 0.84 |  |
| *Asymmetrical lung injury* | | | | | | | | | | | | | | | | |
| Ventilation |  | 5.09  (4.44 - 6.46) |  | 3.50  (3.38 - 6.28) |  | 8.39  (4.27-14.52) |  | **0.03 *** |  |  | 2.90  (1.75 - 3.46) |  | 4.86  (4.26 - 7.54) |  | **0.01*** |  |
| Perfusion |  | 5.74  (3.18 - 6.77) |  | 5.30  (3.25 - 7.04) |  | 8.92  (4.23 -13.60) |  | **0.04 *** |  |  | 3.61  (2.08 - 4.60) |  | 7.74  (4.63 - 8.12) |  | **0.01*** |  |
|  | | | | | | | | | | | | | | | | |
| **Peak** | | | | | | | | | | | | | | | | |
| *Bilateral lung injury* | | | | | | | | | | | | | | | | |
| Ventilation |  | 33.15  (30.84-34.59) |  | 21.39  (14.18 - 33.03) |  | 33.08  (29.86 - 42.14) |  | **0.02** * |  |  | 12.86  (7.40 - 34.05) |  | 16.02  (15.44 - 20.12) |  | 0.44 |  |
| Perfusion |  | 31.04  (25.15-33.15) |  | 30.80  (25-61 - 39.81) |  | 38.69  (33.70 -41.86) |  | **0.04** * |  |  | 27.16  (17.04 - 43.26) |  | 33.20  (19.23 - 26.70) |  | 0.31 |  |
| *Asymmetrical lung injury* | | | | | | | | | | | | | | | | |
| Ventilation |  | 27.13  (22.44 -34.22) |  | 19.46  (14.48 - 27.60) |  | 40.53  (25.09 - 64.19) |  | **0.03** * |  |  | 10.43  (7.35 - 17.55) |  | 26.11  (21.41 -31.50) |  | **0.01 *** |  |
| Perfusion |  | 31.76  (23.46 - 36.70) |  | 28.50  (21.68 - 37.30) |  | 47.38  (26.79 - 65.14) |  | **0.04** * |  |  | 21.77  (15.64 - 25.16) |  | 39.68  (26.74 - 43.42) |  | **0.01 *** |  |
|  | | | | | | | | | | | | | | | | |

Values reported as median (25th quartile - 75th quartile). Two-sided Wilcoxon signed-rank test to test the null hypothesis that the compared data sets (i.e., without iNO vs with iNO) derive from the same continuous distribution. *p<0.05. Abbreviations: PEEP: positive end-expiratory pressure; AOP: airway opening pressure; iNO: inhaled nitric oxide; IQR: interquartile range.

**Table E4.** Lung compartments analysis for each studied lung condition. Percentage of pixels. (compared to sum of all ventilated and/or perfused pixels) belonging to each lung compartment and lung condition.

| **Lung compartment** |  | **Healthy Lung**  **(PEEP 5cmH_2_O)** |  | **PEEP above AOP** | | | | | |  | **PEEP below AOP** | | | | | | |
| --- | --- | --- | --- | --- | --- | --- | --- | --- | --- | --- | --- | --- | --- | --- | --- | --- | --- |
|  |  |  |  | **Without iNO** |  | **With iNO** |  | **Signed rank (without vs with iNO)** |  |  | **Without iNO** |  | **With iNO** |  | **Signed rank (without vs with iNO)** |  |  |
|  |  | median  (IQR) |  | median  (IQR) |  | median  (IQR) |  | p |  |  | median  (IQR) |  | median  (IQR) |  | p |  |  |
| ***Bilateral lung injury*** | | | | | | | | | | | | | | | | | |
| Low V/Q and Shunt (V/Q ≤ 0.5) |  | 45  (35 - 53) |  | 62  (53 - 80) |  | 38  (13 - 40) |  | **0.03*** |  |  | 87  (45 - 109) |  | 93  (50 - 98) |  | 1.00 |  |  |
| Normal V/Q (V/Q 0.5 to 2) |  | 332  (266-363) |  | 193  (136 -237) |  | 207  (232 - 303) |  | **0.04*** |  |  | 144  (104 - 200) |  | 213  (184 - 229) |  | 1.00 |  |  |
| High V/Q and Dead Space (V/Q ≥ 2) |  | 53  (35 - 65) |  | 107  (92 - 159) |  | 72  (42 - 85) |  | **0.04*** |  |  | 136  (89 - 154) |  | 90  (87-100) |  | **0.03*** |  |  |
|  | | | | | | | | | | | | | | | | | |
| ***Asymmetrical lung injury*** | | | | | | | | | | | | | | | | | |
| Low V/Q and Shunt (V/Q ≤ 0.5) |  | 43  (21 - 50) |  | 67  (26 - 78) |  | 12  (9 - 27) |  | **0.02*** |  |  | 74  (67 - 75) |  | 36  (14 - 54) |  | **0.02*** |  |  |
| Normal V/Q (V/Q 0.5 to 2) |  | 283  (257-309) |  | 165  (112 - 207) |  | 228  (215 - 256) |  | **0.03*** |  |  | 69  (46 - 135) |  | 184  (153 - 218) |  | **0.02*** |  |  |
| Low V/Q and Dead Space (V/Q ≥ 2) |  | 70  (53 - 112) |  | 117  (84 - 134) |  | 56  (39 - 90) |  | **0.03*** |  |  | 135  (93 - 159) |  | 79  (59 - 105) |  | **0.02*** |  |  |

Values reported as median (25th quartile - 75th quartile). Two-sided Wilcoxon signed rank test (α=0.05) to test the null hypothesis that the compared data sets (i.e., without iNO vs with iNO) derive from the same continuous distribution. *: to mark significant differences. Abbreviations: PEEP: positive end-expiratory pressure; AOP: airway opening pressure; iNO: inhaled nitric oxide; signrank: two-sided Wilcoxon signed rank test; IQR: interquartile range.

**Table E5.** Lung compartments analysis for each studied lung condition. Absolute number of pixels for each lung compartment and lung condition.

| **Lung compartment** |  | **Healthy Lung**  **(PEEP 5cmH_2_O)** |  | **PEEP above AOP** | | | | | | | | | | | | |  | | | **PEEP below AOP** | | | | | | | | | | | | | | | |  |  |
| --- | --- | --- | --- | --- | --- | --- | --- | --- | --- | --- | --- | --- | --- | --- | --- | --- | --- | --- | --- | --- | --- | --- | --- | --- | --- | --- | --- | --- | --- | --- | --- | --- | --- | --- | --- | --- | --- |
|  |  |  |  | **without iNO** |  | **with iNO** | | | |  | | **Signed rank (α=0.05) without vs with iNO** | | |  | | |  | | | **without iNO** | | |  | | **with iNO** | | | |  | | **Signed rank (α=0.05) without vs with iNO** | |  | | |  |
|  |  | Median  (IQR) |  | Median  (IQR) |  | Median  (IQR) | | | |  | | p | | |  | | |  | | | Median  (IQR) | | |  | | Median  (IQR) | | | |  | | p | |  | | |  |
| ***Bilateral lung injury*** | | | | | | | | | | | | | | | | | | | | | | | | | | | | | | | | | | | |  |  |
| Low V/Q and Shunt (V/Q ≤ 0.5) |  | 10  (8 - 14) |  | 16  (13 - 19) |  | | | 9  (4 - 12) | | |  | | **0.03*** | | |  | | |  | | | 25  (16 - 30) | | |  | | 23  (14 - 27) | | | |  | | 0.68 | |  | | |
| Normal V/Q (V/Q 0.5 to 2) |  | 80  (69 -85) |  | 51  (38 - 66) |  | | | 70  (65 - 85) | | |  | | **0.03*** | | |  | | |  | | | 41  (28 - 53) | | |  | | 52  (50 - 64) | | | |  | | 0.68 | |  | | |
| High V/Q and Dead Space (V/Q ≥ 2) |  | 13  (8 – 17) |  | 30  (24 – 43) |  | | | 19  (14 – 23) | | |  | | **0.03*** | | |  | | |  | | | 31  (28 – 42) | | |  | | 22  (20 – 32) | | | |  | | 0.09 | |  | | |
|  |  |  |  |  |  | |  | |  | | | | |  | |  | | |  | | |  |  | | | | |  |  | | | |  | |  | | |
| ***Asymmetrical lung injury*** | | | | | | | | | | | | | | | | | | | | | | | | | | | | | | | | | | | |  |  |
| Low V/Q and Shunt (V/Q ≤ 0.5) |  | 10  (5 - 12) |  | 19  (8 - 26) |  | 5  (3 - 9) | | | |  | | **0.02*** | | |  | | |  | | | 25  (23 - 27) | | |  | | 11  (5 - 17) | | | |  | | **0.02*** | |  | | |  |
| Normal V/Q (V/Q 0.5 to 2) |  | 70  (68 - 73) |  | 45  (40 - 60) |  | 77  (67 - 85) | | | |  | | **0.03*** | | |  | | |  | | | 23  (18 - 47) | | |  | | 65  (51 - 67) | | | |  | | **0.02*** | |  | | |  |
| High V/Q and Dead Space (V/Q ≥ 2) |  | 16  (15 - 27) |  | 33  (30 - 36) |  | 20  (12 - 27) | | | |  | | **0.01*** | | |  | | |  | | | 45  (33 - 55) | | |  | | 28  (20 - 32) | | | |  | | **0.02*** | |  | | |  |

Values reported as median (25th quartile - 75th quartile). Two-sided Wilcoxon signed-rank test to test the null hypothesis that the compared data sets (i.e., without iNO vs with iNO) derive from the same continuous distribution. *p<0.05. Abbreviations: PEEP: positive end-expiratory pressure; AOP: airway opening pressure; iNO: inhaled nitric oxide; IQR: interquartile range.
