# Supplementary figures and images for: Impact of airway closure and lung collapse on inhaled nitric oxide effect in acute lung injury: an experimental study
Source: Ann Intensive Care. 2024 Sep 23;14:149. doi: 10.1186/s13613-024-01378-z (PMC11420414; doi:10.1186/s13613-024-01378-z)

## Slide 1
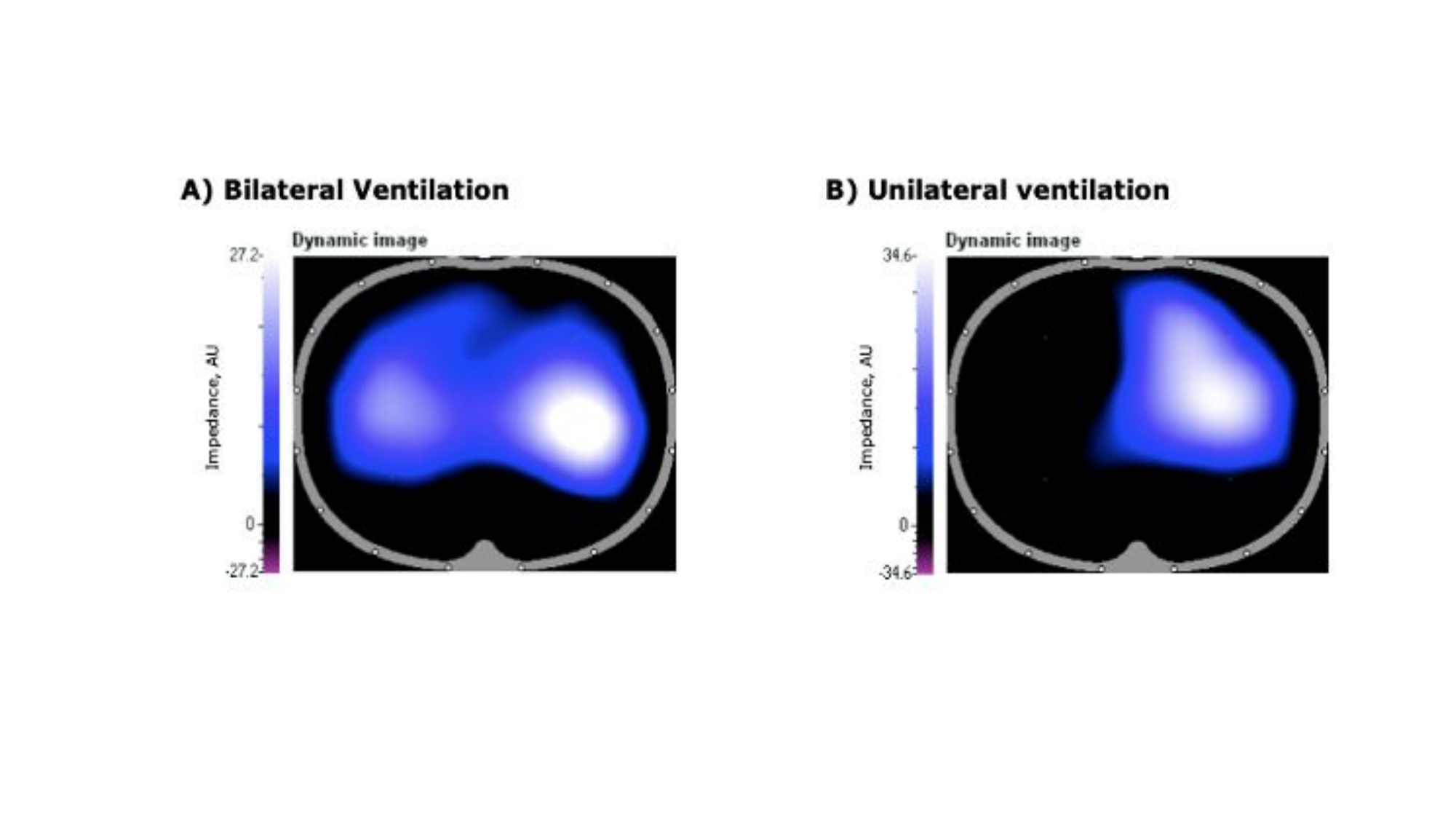

## Slide 2
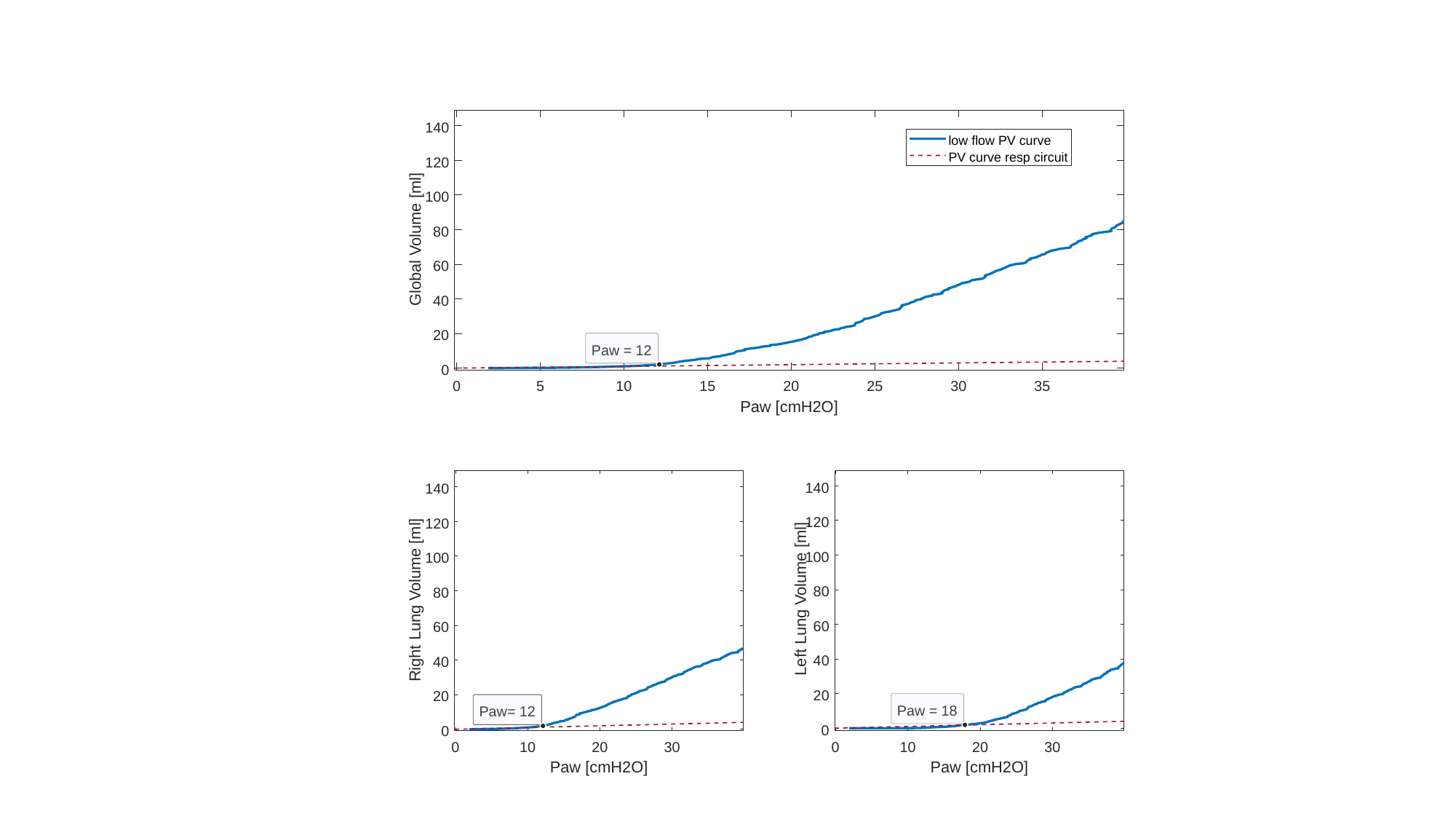

## Slide 3
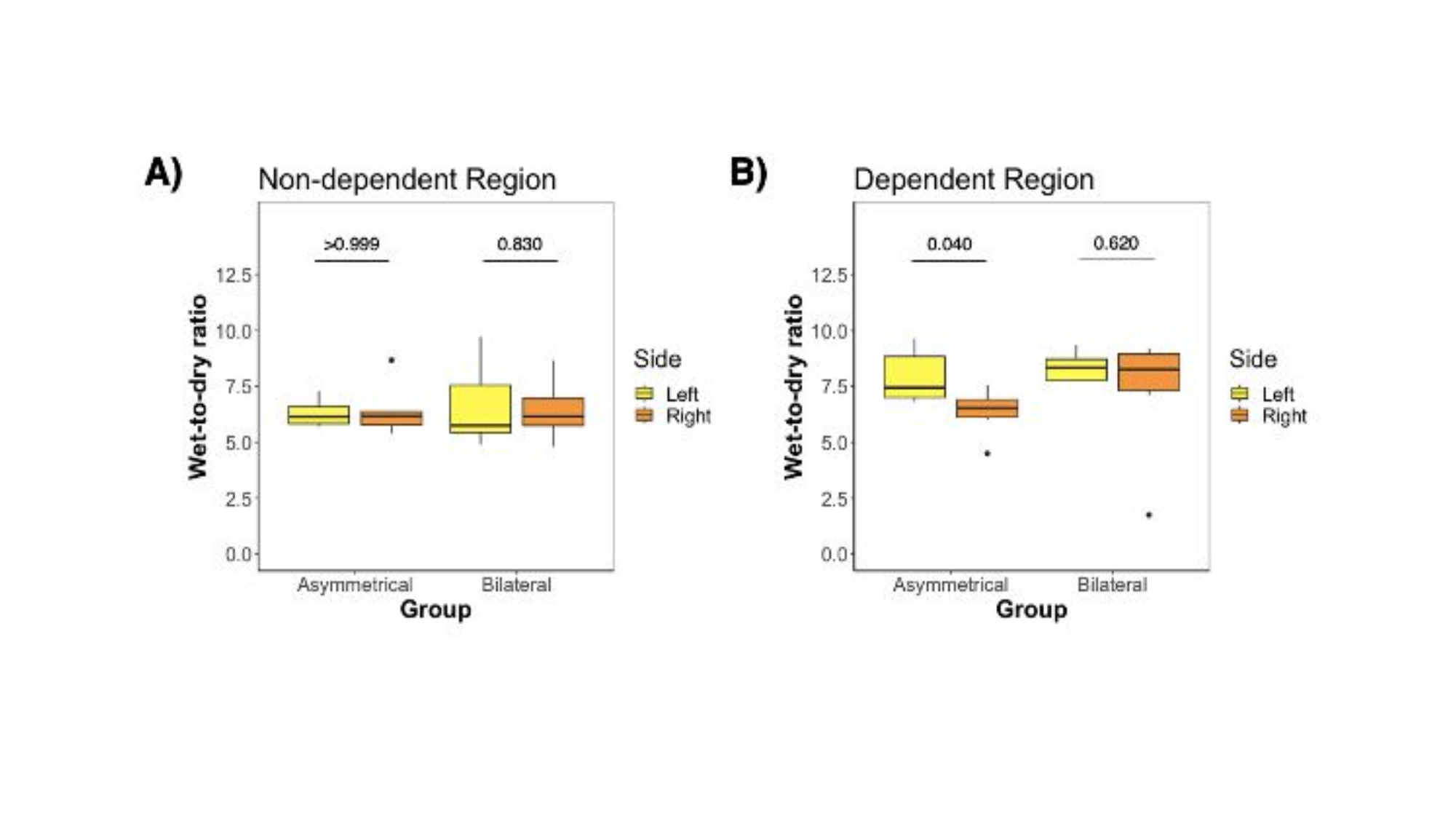

## Slide 4
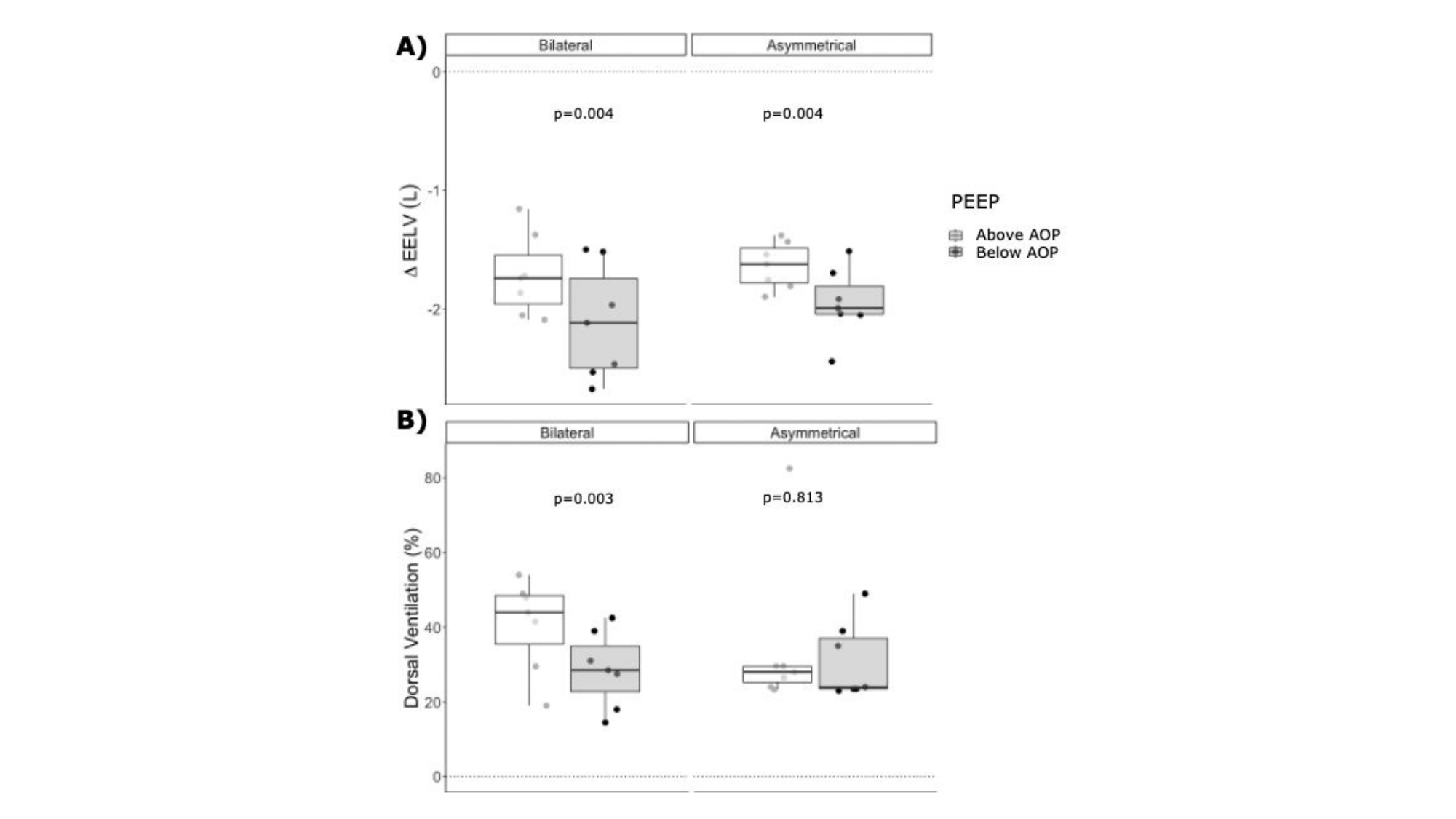

## Slide 5
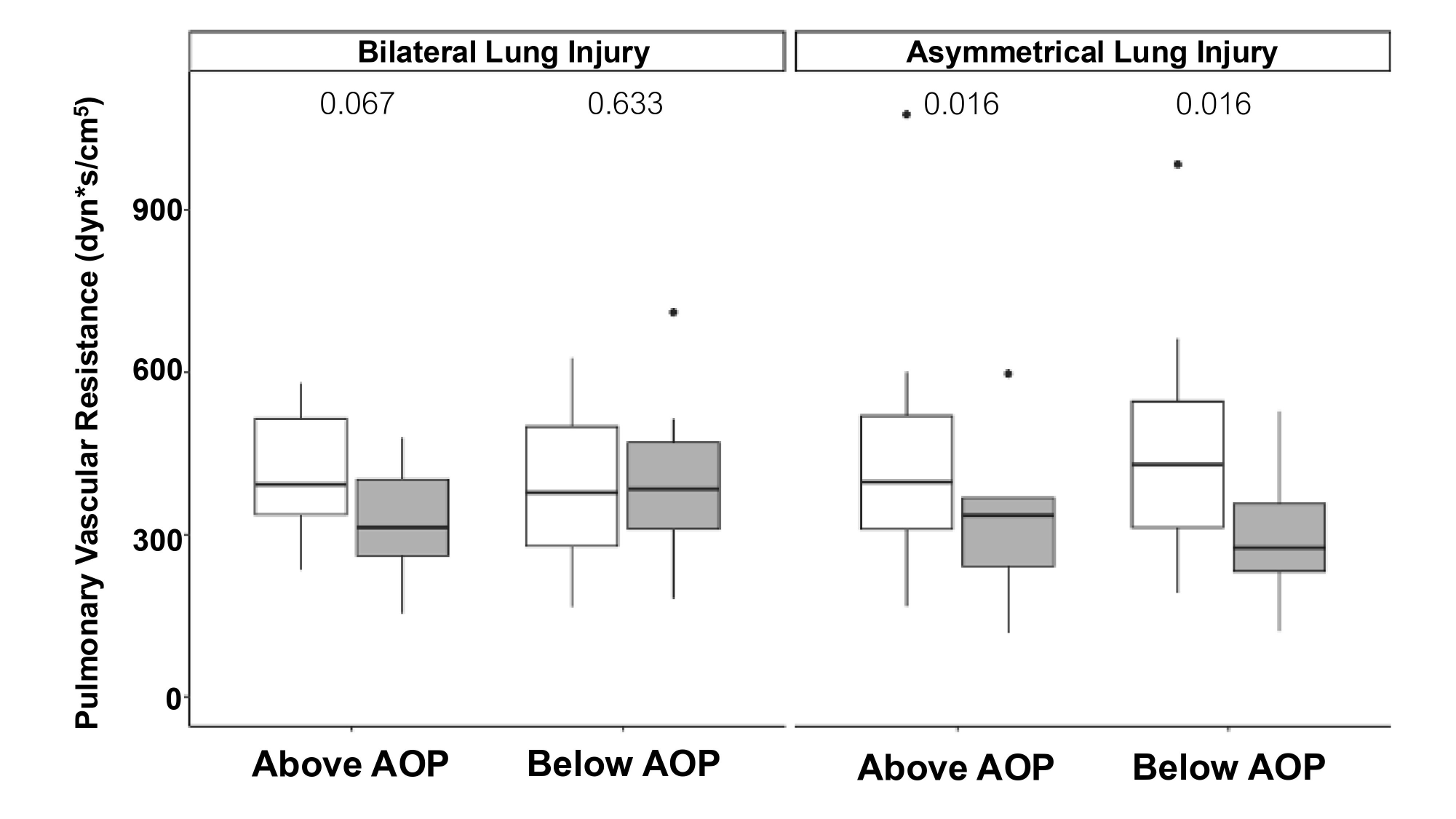

Supplement: Supplementary file 2 — Supplementary Material 2: Figure E1. Representative case of lung ventilation monitored by Electrical Impedance Tomography (EIT) in the Asymmetrical Group. (A) ventilation of both lungs with single-lumen tube before lung injury; (B) ventilation of the left lung only, with double-lumen tube, after 2-hit lung injury. In the images ventilated areas are presented in blue-to-white scale and non-ventilated areas in black. Figure E2. A representative case of low flow PV curves and airway opening pressure (AOP) defection. PV curves performed after lung injury. Paw on the x-axis, volume [ml] estimated based on the correspondence between inspiratory impedance changes and inspiratory volume at the end of the PV curve maneuver. Data tips indicate the Paw corresponding to AOP for 1) the global respiratory system (right + left lung) - plot above; 2) the right lung and 3) the left lung – plots below. Figure E3. Lung wet-to-dry ratio. A) Non-dependent lung regions. B) Dependent lung regions. p-values are from univariate analysis (t-test or Mann-Whitney test, according to distribution). Figure E3. Lung wet-to-dry ratio. A) Non-dependent lung regions. B) Dependent lung regions. p-values are from univariate analysis (t-test or Mann-Whitney test, according to distribution). Figure E4. Changes in (A) end-expiratory lung volume (?EELV) and (B) dorsal ventilation consequent to changes in positive end-expiratory pressure (PEEP). PEEP above airway opening pressure (AOP) reported in white versus PEEP below AOP reported in gray. [file 13613_2024_1378_MOESM2_ESM.pptx]
